# Radiomic signatures reveal multiscale intratumor heterogeneity associated with tissue tolerance and survival in re-irradiated nasopharyngeal carcinoma: a multicenter study

**DOI:** 10.1186/s12916-023-03164-3

**Published:** 2023-11-27

**Authors:** Ting Liu, Di Dong, Xun Zhao, Xiao-Min Ou, Jun-Lin Yi, Jian Guan, Ye Zhang, Lv Xiao-Fei, Chuan-Miao Xie, Dong-Hua Luo, Rui Sun, Qiu-Yan Chen, Lv Xing, Shan-Shan Guo, Li-Ting Liu, Da-Feng Lin, Yan-Zhou Chen, Jie-Yi Lin, Mei-Juan Luo, Wen-Bin Yan, Mei-Lin He, Meng-Yuan Mao, Man-Yi Zhu, Wen-Hui Chen, Bo-Wen Shen, Shi-Qian Wang, Hai-Lin Li, Lian-Zhen Zhong, Chao-Su Hu, De-Hua Wu, Hai-Qiang Mai, Jie Tian, Lin-Quan Tang

**Affiliations:** 1 Sun Yat-Sen University Cancer CenterState Key Laboratory of Oncology in South ChinaCollaborative Innovation Center for Cancer Medicine, Guangdong Key Laboratory of Nasopharyngeal Carcinoma Diagnosis and Therapy, Guangzhou, China; 2 Department of Nasopharyngeal Carcinoma, Sun Yat-Sen University Cancer Center, 651 Dongfeng Road East, Guangzhou, 510060 China; 3 Breast Disease Center, The First Affiliated Hospital of Sun Yat-Sen University, Guangzhou, China; 4 CAS Key Laboratory of Molecular Imaging, Institute of Automation, Chinese Academy of Sciences, Beijing, 100190 China; 5 School of Artificial Intelligence, University of Chinese Academy of Sciences, Beijing, China; 6 Department of Radiation Oncology, Fudan University Shanghai Cancer Center, Shanghai, China; 7 Department of Oncology, Shanghai Medical College, Fudan University, Shanghai, China; 8 Department of Radiation Oncology, National Cancer Center/National Clinical Research Center for Cancer/Cancer Hospital, Chinese Academy of Medical Sciences and Peking Union Medical College, Beijing, China; 9 Department of Radiation Oncology, Nanfang Hospital, Southern Medical University, Guangzhou, China; 10 Department of Radiology, Sun Yat-Sen University Cancer Center, Guangzhou, China; 11 Department of Oncology, the First Affiliated Hospital, Jinan University, Guangzhou, China; 12 Zhongshan School of Medicine, Sun Yat-Sen University, Guangzhou, China; 13 Beijing Advanced Innovation Center for Big Data-Based Precision Medicine, Beihang University, Beijing, China

**Keywords:** Recurrent nasopharyngeal carcinoma, Re-radiotherapy, Nasopharyngeal necrosis, Radiomics

## Abstract

**Background:**

Post-radiation nasopharyngeal necrosis (PRNN) is a severe adverse event following re-radiotherapy for patients with locally recurrent nasopharyngeal carcinoma (LRNPC) and associated with decreased survival. Biological heterogeneity in recurrent tumors contributes to the different risks of PRNN. Radiomics can be used to mine high-throughput non-invasive image features to predict clinical outcomes and capture underlying biological functions. We aimed to develop a radiogenomic signature for the pre-treatment prediction of PRNN to guide re-radiotherapy in patients with LRNPC.

**Methods:**

This multicenter study included 761 re-irradiated patients with LRNPC at four centers in NPC endemic area and divided them into training, internal validation, and external validation cohorts. We built a machine learning (random forest) radiomic signature based on the pre-treatment multiparametric magnetic resonance images for predicting PRNN following re-radiotherapy. We comprehensively assessed the performance of the radiomic signature. Transcriptomic sequencing and gene set enrichment analyses were conducted to identify the associated biological processes.

**Results:**

The radiomic signature showed discrimination of 1-year PRNN in the training, internal validation, and external validation cohorts (area under the curve (AUC) 0.713–0.756). Stratified by a cutoff score of 0.735, patients with high-risk signature had higher incidences of PRNN than patients with low-risk signature (1-year PRNN rates 42.2–62.5% vs. 16.3–18.8%, *P* < 0.001). The signature significantly outperformed the clinical model (*P* < 0.05) and was generalizable across different centers, imaging parameters, and patient subgroups. The radiomic signature had prognostic value concerning its correlation with PRNN-related deaths (hazard ratio (HR) 3.07–6.75, *P* < 0.001) and all causes of deaths (HR 1.53–2.30, *P* < 0.01). Radiogenomics analyses revealed associations between the radiomic signature and signaling pathways involved in tissue fibrosis and vascularity.

**Conclusions:**

We present a radiomic signature for the individualized risk assessment of PRNN following re-radiotherapy, which may serve as a noninvasive radio-biomarker of radiation injury-associated processes and a useful clinical tool to personalize treatment recommendations for patients with LANPC.

**Supplementary Information:**

The online version contains supplementary material available at 10.1186/s12916-023-03164-3.

## Background

Nasopharyngeal carcinoma (NPC) is an endemic head and neck cancer in southeast Asia [1, 2]. Despite improvements in radiation dosimetry via the implementation of intensity modulated radiotherapy (IMRT) in the primary treatment of NPC, 10–15% of patients develop local recurrence [3]. Salvage re-radiotherapy and nasopharyngectomy are potentially curative approaches for locally recurrent NPC (LRNPC) [4]; however, the feasibility of surgery is often challenged by the ability to obtain a sufficient margin in the confined anatomical space of nasopharynx [5].

**Fig. 1:**
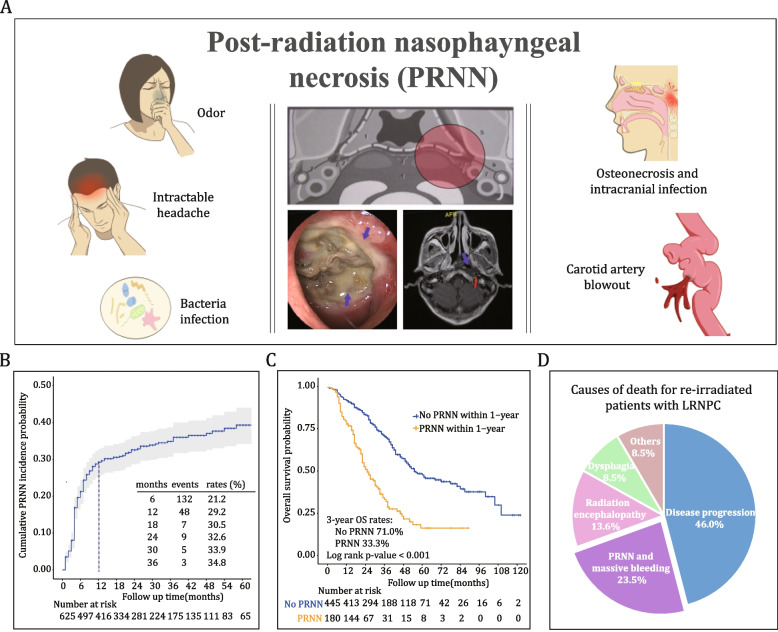
Clinical characteristics of post-radiation nasopharyngeal necrosis. **A** Typical endoscopic and magnetic resonance imaging manifestations of post-radiation nasopharyngeal necrosis (PRNN), along with the associated symptoms and complications. **B** Incidences of PRNN following re-radiotherapy in 625 patients with locally recurrent nasopharyngeal carcinoma (LRNPC) recruited from the Sun Yat-sen University Cancer Centre. Most events occurred within 1 year of re-radiotherapy. **C** Kaplan–Meier curve showing that PRNN significantly impaired overall survival (OS). **D** PRNN and massive bleeding are leading causes of death for re-irradiated patients with LRNPC

An important consideration regarding re-radiotherapy is the risk and severity of radiation-induced toxicities. According to previous studies, up to 50% of re-irradiated patients died from grade 5 toxicities, with the most common toxicities being nasopharyngeal necrosis and massive bleeding, followed by temporal lobe necrosis and dysphagia [6, 7]. Post-radiation nasopharyngeal necrosis (PRNN) is a severe adverse event of re-radiotherapy with a reported incidence of 28–41% [6, 8]. Owing to its close proximity to skull base and vital arteries, PRNN causes odor and intractable headache, followed by skull base osteonecrosis, infection, carotid artery involvement, and massive bleeding, which greatly compromise patients’ quality of life and long-term survival (Fig. 1, Additional file 1: Figure S1) [9–11]. Due to the accumulated doses of two courses of radiotherapy, PRNN following re-radiotherapy is typically severe, with an early onset and rapid progression, and is refractory to treatment. PRNN is a dose-limiting factor of re-radiotherapy for LRNPC. Accurate pre-treatment assessment of the risk of PRNN is crucial to patient selection and tailoring of re-radiotherapy regimens.

Some clinical factors are associated with the development of PRNN following re-radiotherapy [12]; however, a pure clinical model showed limited predictive value [13], and the underlying pathophysiology is largely unknown. Magnetic resonance imaging (MRI) is commonly used for the staging process and pre-radiotherapy workup of LRNPC in clinical practice [14]. An MRI-based signature that detects encrypted information in the recurrent region regarding tissue tolerance to re-irradiation could have important clinical implications for PRNN prediction. Advances in radiomics and machine learning enable the extraction of large amounts of quantitative data from medical imaging and thus allow the identification of clinically significant imaging patterns that cannot be recognized by a human reader [15–18]. Linking such imaging patterns to underlying biological processes using a radiogenomics approach may provide novel insights into the implications of radiomic signature and disease mechanisms [19, 20].

Previously, two studies looked to predict PRNN in NPC [12, 21]. Both studies used logistic regression method to construct pure clinical models; the related variables included sex, age, tumor volume, radiation doses and techniques, etc. Only one study quantitatively assessed the performance of their model; they reported an accuracy value of 0.78 [21]. However, most of the patients included in their study were newly diagnosed with NPC and treated with the first course of radiotherapy (re-irradiated patients only accounted for 3.4%). Predicting PRNN specifically for re-irradiated patients would be more clinically significant but more challenging. There are no published studies investigating the role of radiomic features in the prediction of PRNN. In this multicenter study, we aimed to develop and validate a machine learning-derived radiomic model based on the pre-treatment MR images to predict PRNN risk following curative re-radiotherapy for LRNPC and to explore the potential biological processes associated with the radiomic signature.

## Methods

### Study cohorts

**Fig. 2:**
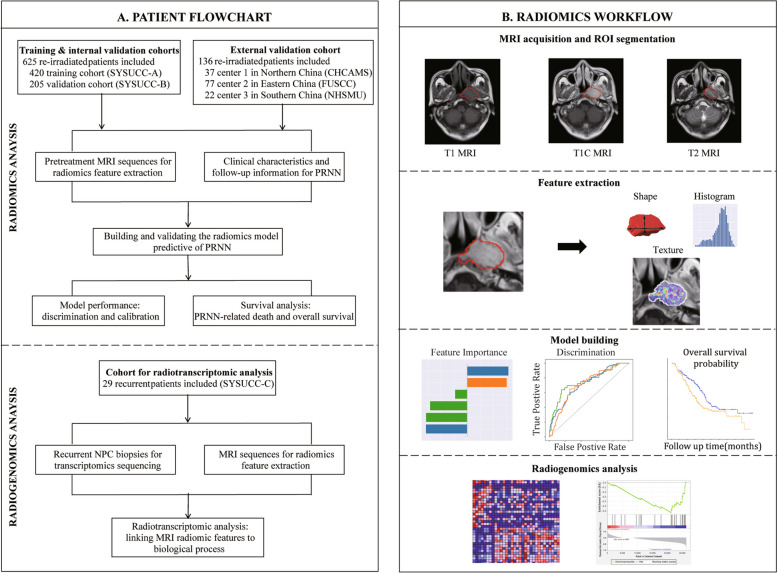
Study flowchart. **A** Multicentre cohorts of patients with locally recurrent nasopharyngeal carcinoma (LRNPC) who were treated with re-radiotherapy. **B** Radiomics modelling for the prediction of post-radiation nasopharyngeal necrosis (PRNN) based on pre-treatment head and neck magnetic resonance imaging (MRI). SYSUCC Sun Yat-sen University Cancer Centre, CHCAMS Cancer Hospital Chinese Academy of Medical Sciences, FUSCC Fudan University Shanghai Cancer Centre, NHSMU Nanfang Hospital of Southern Medical University, ROI region-of-interest

The radiomics study included multicenter cohorts of 761 patients with LRNPC who received curative re-radiotherapy between 2011 and 2019 from four medical centers (Fig. 2). Patients recruited from the Sun Yat-sen University Cancer Center (SYSUCC) were randomly split in 7:3 to construct the training (420 patients) and internal validation (205 patients) cohorts; a pooled cohort from the remaining three centers constituted the external validation cohort (136 patients). The radiogenomics study included 29 patients from the SYSUCC cohort who were diagnosed with LRNPC and underwent endoscopic biopsies (Fig. 2). The inclusion criteria, exclusion criteria, treatment protocols, and patient characteristics are presented in Additional file 1: Methods A1, A2, A3 and Table 1. This study was approved by the clinical research committee of the study centers, and written informed consent was retrieved from all included patients.

**Table 1:** Patient characteristics

	Training cohort	Internal validation cohort	External validation cohort	***P*** value
Sample size, patients	420	205	136	
PRNN events within 1-year (%)	120 (28.6)	60 (29.3)	36 (26.5)	0.847
PRNN events in total (%)	148 (35.2)	66 (32.2)	45 (33.1)	0.728
Median age at recurrence, years (IQR)	47.0 (40.8–53.0)	48.0 (42.0–55.0)	49.0 (41.0–54.0)	0.228
Sex (%)				0.759
Male	316 (75.2)	152 (74.1)	98 (72.1)	
Female	104 (24.8)	53 (25.9)	38 (27.9)	
Median BMI, kg/m2 (IQR)	22.3 (20.4–24.2)	22.2 (19.8–24.2)	22.1 (20.0–24.5)	0.814
History of diabetes (%)	14 (3.3)	4 (2.0)	9 (6.6)	0.070
History of hypertension (%)	31 (7.4)	14 (6.8)	5 (3.7)	0.312
Recurrent T category (%)^a^				< 0.001
rT1	21 (5.0)	7 (3.4)	18 (13.2)	
rT2	46 (11.0)	24 (11.7)	32 (23.5)	
rT3	208 (49.5)	108 (52.7)	58 (42.6)	
rT4	145 (34.5)	66 (32.2)	28 (20.6)	
Synchronous nodal recurrence (%)	128 (30.5)	61 (29.8)	46 (33.8)	0.703
Recurrent histology (%)				< 0.001
WHO III	397 (94.5)	192 (93.7)	108 (79.4)	
WHO II	4 (1.0)	4 (2.0)	3 (2.2)	
WHO I	19 (4.5)	9 (4.4)	25 (18.4)	
Median disease-free interval, months (IQR)	34.0 (20.0–57.0)	34.0 (22.0–65.0)	36.5 (20.0–59.0)	0.606
Median GTV_recurrence_, cm3 (IQR)	40.6 (23.7–70.0)	35.7 (20.0–62.0)	26.0 (18.0–51.0)	< 0.001
Re-radiotherapy type (%)				0.004
IMRT	375 (89.3)	187 (91.2)	134 (98.5)	
Helical tomotherapy	45 (10.7)	18 (8.8)	2 (1.5)	
Median re-irradiation dose, Gy (IQR)^b^	61.1 (60.0–64.0)	61.1 (59.7–64.0)	64.0 (61.1–64.0)	< 0.001
Re-irradiation dose (%)^b^				< 0.001
< 64 Gy	281 (66.9)	137 (66.8)	40 (29.4)	
≥ 64 Gy, < 68 Gy	119 (28.3)	56 (27.3)	87 (64.0)	
≥ 68 Gy	20 (4.8)	12 (5.9)	9 (6.6)	
Median accumulated GTV dose, Gy (IQR)^b^	131.1 (129.7–134.0)	131.1 (129.7–134.0)	134.0 (130.0–134.0)	0.013
Accumulated GTV dose (%)^b^				< 0.001
< 134 Gy	295 (70.2)	143 (69.8)	61 (44.9)	
≥ 134 Gy, < 140 Gy	106 (25.2)	53 (25.9)	71 (52.2)	
≥ 140 Gy	19 (4.5)	9 (4.4)	4 (2.9)	
Combination with chemotherapy (%)	291 (69.3)	137 (66.8)	91 (66.9)	0.775
Median hemoglobin level, g/L (IQR)	137.0 (126.0–148.0)	136.0 (127.0–148.0)	136.5 (128.0–145.3)	0.959

*IQR* interquartile range, *BMI* body mass index, *GTV* gross tumor volume, *IMRT* intensity modulated radiotherapy

^a^Patients were restaged according to the 8th American Joint Committee on Cancer Union for International Cancer Control (UICC/AJCC) staging system

^b^Doses were converted to equivalent dose in 2-Gy fraction (EQD2) values using the following equation: total dose * (dose per fraction + 10) / (2 + 10), where 10 = a/b ratio for head and neck cancer

### Clinical outcomes and follow up

The primary outcome was PRNN within 1 year of re-radiotherapy. We chose the time window of 1-year post-radiotherapy to observe PRNN because this is the period that most re-irradiated patients survived and most PRNN events occurred (Fig. 1). The secondary outcomes included grade 5 necrosis-free survival (G5-NFS, defined as the interval from the first day of re-radiotherapy to death from any PRNN-related cause), and overall survival (OS, defined as the interval from the first day of re-radiotherapy to death from any cause). PRNN was diagnosed according to clinical symptoms, nasopharyngeal endoscopy, MRI, and pathological examinations (Additional file 1: Methods A4, Figure S2). All patients were followed up according to the routine practice of the study centers for ≥ 12 months after re-radiotherapy or until death.

### MR image acquisition and segmentation

Pre-treatment head and neck MR images were retrieved from the four medical centers, manually segmented by experienced radiation oncologists, and then transferred to the Institute of Automation, Chinese Academy of Sciences (Peking, China) for analysis. MRI scanning comprised at least three series of axial T1-weighted, T2-weighted, and contrast-enhanced T1-weighted images. The MRI parameters are shown in Additional file 1: Table S1. Recurrent tumors localized in the nasopharynx were selected as the main region-of-interest (ROI_nx). ROI segmentation was performed on three axial MRI sequences by professional radiation oncologists in the four medical centers using the ITK-SNAP software (version 3.8.0; www.itksnap.org). After 4 weeks, 50 patients in the training cohort were randomly selected and their ROIs were segmented again by the same oncologist and another professional radiation oncologist to assess the intra-reader and inter-reader reproducibility of radiomic features. Details regarding ROI segmentation are provided in Additional file 1: Figure S3 and Methods A5.

### Radiomic features extraction

All images were resampled to a voxel resolution of 0.5 × 0.5 × 3.0 mm for quantitative feature extraction. Radiomic features were calculated on three MR image series using the PyRadiomics package of Python 3.7.5 [22] (Additional file 1: Methods A6). The extracted features consisted of three types: first order statistics, shape, and texture (Gray Level Co-occurrence Matrix [GLCM], Gray Level Dependence Matrix [GLDM], Gray Level Run-Length Matrix [GLRLM], and Gray Level Size Zone Matrix [GLSZM]). A total of 2106 features (702 features in each MRI series) were extracted for ROI analysis.

### Radiomic signature building

To avoid model overfitting and improve performance, feature selection was performed in the training cohort as follows (Additional file 1: Methods A7): first, we used intra/inter-class correlation coefficients (ICCs) to assess the reproducibility of extracted radiomic features based on re-segmented data. Only stable features with ICCs of > 0.7 were selected. Second, three feature ranking methods which included univariate logistic regression, lasso regression, and linear support vector machines (SVM) binary classifier were adopted to select the 100 most important features in each series for further analysis. Third, a fine selection approach was applied to select the most representative features to generate candidate classification models. We applied a grid search strategy to determine the best number of features to be remained and filtered the selected features recursively using a recursive feature elimination (RFE) approach based on the model’s accuracy to predict PRNN on repeated fivefold cross validation. We limited the correlation coefficient between features to < 0.7 in RFE to reduce redundancy. In this study, three widely-used modelling algorithms were used to generate models, e.g., multivariate logistic regression, linear SVM, and random forest. Finally, the model that achieved the highest performance in the training cohort was selected. The model output (namely the probability of belonging to the 1-year PRNN vs. non-PRNN group) was denoted as the radiomic signature. The constructed radiomic signature was then applied to the internal and external validation cohorts.

### Exploratory analysis

We compared the radiomic signature to routine clinical variables in terms of the ability to predict PRNN (Additional file 1: Methods A8). We evaluated whether the addition of clinical variables to radiomic signature would result in improved prediction.

### Model performance

The accuracy of candidate models to predict 1-year PRNN was compared using the area under the receiver operating characteristic (ROC) curve (AUC). For creating a confusion matrix based on the final signature for PRNN prediction, a cutoff was selected on the training data using Youden index and transferred to the validation data. Other analyses were conducted to assess the generalizability, calibration, and clinical utility of the radiomic signature (Additional file 1: Methods A9). Patients were stratified into two subgroups according to the risks of PRNN based on a cutoff value of the radiomic signature that stratified the training cohort with the most significant log-rank test. The prognostic value of the radiomic signature for (i) PRNN, (ii) G5-NFS, and (iii) OS was analyzed.

### Biological correlation

The constructed radiomic signature was applied to the 29 patients included in radiogenomics study; the corresponding transcriptomic data were obtained from tissue samples (Additional file 1: Methods A10). Gene set enrichment analysis (GSEA) was used to identify the potential gene ontology (GO) based biological processes associated with the radiomic signature [23]. Single-sample GSEA (ssGSEA) was applied to explore the biological implications of the individual radiomic features (Additional file 1: Methods A11) [24].

### Statistical analysis

Patient characteristics were compared using the chi-squared test for categorical variables and Mann–Whitney *U* test for continuous variables. Bootstrapping was used to estimate the 95% confidence intervals (CIs) of the AUC. All statistical analyses were conducted with R 3.6.1, with a two-sided *P*-value < 0.05 to indicate statistical difference. A radiomics quality score tool was applied to assess the methodological quality of this study (Additional file 1: Methods A12) [20].

## Results

### Patient demographics and clinical outcomes

We summarized clinical features of PRNN in Fig. 1 and Additional file 1: Figure S1, concerning its location, symptoms, diagnosis, and outcomes. The clinical characteristics of the included patients are presented in Table 1. With a median follow-up time of 44, 39, and 48 months, respectively, in the training, internal validation, and external validation cohorts, we observed 148 (35.2%), 66 (32.1%), and 45 (33.0%) PRNN events in total. Of these, 120 (81.1%), 60 (90.9%), and 36 (80.0%), respectively, occurred within 1 year of re-radiotherapy. The causes of death for patients with and without PRNN are summarized in Additional file 1: Figure S4 and Table S2.

### Development of a radiomic signature for predicting PRNN

We applied the combinations of different feature selecting and classifying algorithms including logistic regression, lasso regression, support vector machines, and random forest to select the most powerful method for our model (Additional file 1: Table S3). Among the 18 candidate models, we chose random forest as the classifier model and linear SVM as the feature selection model to construct the radiomic signature, which achieved the highest accuracy for predicting PRNN in the training cohort (model hyperparameters presented in Additional file 1: Methods A13). After careful selection, 6 out of the 2106 radiomic features were used to build the model (Additional file 1: Table S4); these features exhibited good independency (Additional file 1: Figure S5). The six radiomic features included one first-order statistic, two shape features, and three texture features (Additional file 1: Table S5, definitions in Methods A13).

**Fig. 3:**
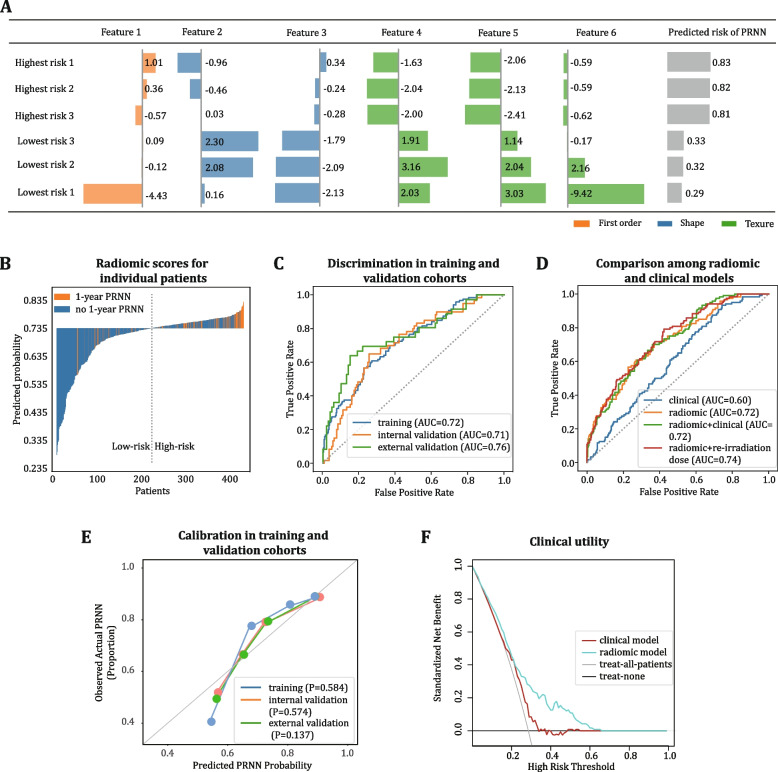
Performance of the radiomic model in predicting post-radiation nasopharyngeal necrosis. **A** Visualization of the six radiomic features included in the signature. The changes in the individual radiomic features in patients with the highest and lowest probability of 1-year post-radiation nasopharyngeal necrosis (PRNN) are presented. **B** Output of the radiomic signature for each patient in the training cohort. **C** Receiver operating characteristic curves of the radiomic signature to predict 1-year PRNN in the training, internal validation, and external validation cohorts. **D** Comparison of the radiomic and clinical models to predict 1-year PRNN in the training cohort. **E** Calibration curve of the PRNN radiomic signature. The significance of calibration was analyzed using Hosmer–Lemeshow goodness-of-fit tests, with the *P*-value shown. **F** Clinical utility of the PRNN radiomic signature to guide re-radiotherapy based on decision curve analysis. With the threshold probability set at > 20%, using the radiomic signature to predict PRNN adds more benefit than either the treat-all-patients strategy or the treat-none strategy; the radiomic signature produces more net benefits than the clinical model. AUC area under the receiver operating characteristic curve

The radiomic signature was computed for all patients (Fig. 3A-B). The signature had AUCs of 0.722 (95% CI 0.676–0.765), 0.713 (95% CI 0.653–0.772), and 0.756 (95% CI 0.673–0.838) for predicting 1-year PRNN in the training, internal validation, and external validation cohorts, respectively (Fig. 3C). At a threshold of 0.732, the signature was able to accurately classify 90/120 PRNN events and 208/300 no PRNN events in the training cohort; similar prediction effect was seen in the internal and external validation cohorts (Additional file 1: Figure S6). The radiomic signature significantly outperformed the individual clinical predictors (AUC of gross tumor volume (GTV)_recurrence_ 0.588, age 0.565, disease-free interval (DFI, defined as the interval between the end of the first course of radiotherapy and diagnosis of recurrence) 0.552, sex 0.519, and re-irradiation dose 0.496 (Additional file 1: Table S6), and combined clinical model (AUC 0.600, *P* < 0.05, Fig. 3D, Additional file 1: Table S7). After adding a dosimetric factor of the re-irradiation dose to the radiomic signature, the discriminative ability of the model slightly improved (AUC 0.741) but not significantly. The addition of other variables including sex, age, DFI, and GTV_recurrence_ did not provide incremental value.

The radiomic signature displayed generalizability across different medical centers, MRI parameters, and patient subgroups (AUC 0.671–0.888, Additional file 1: Table S8 and Table S9). The calibration curve demonstrated good concordance between the predicted 1-year PRNN probability and actual incidence (Fig. 3E). Decision curve analysis demonstrated that the utility of the signature to guide re-radiotherapy would be more beneficial than the treat-all-patients and treat-none strategy (Fig. 3F).

**Fig. 4:**
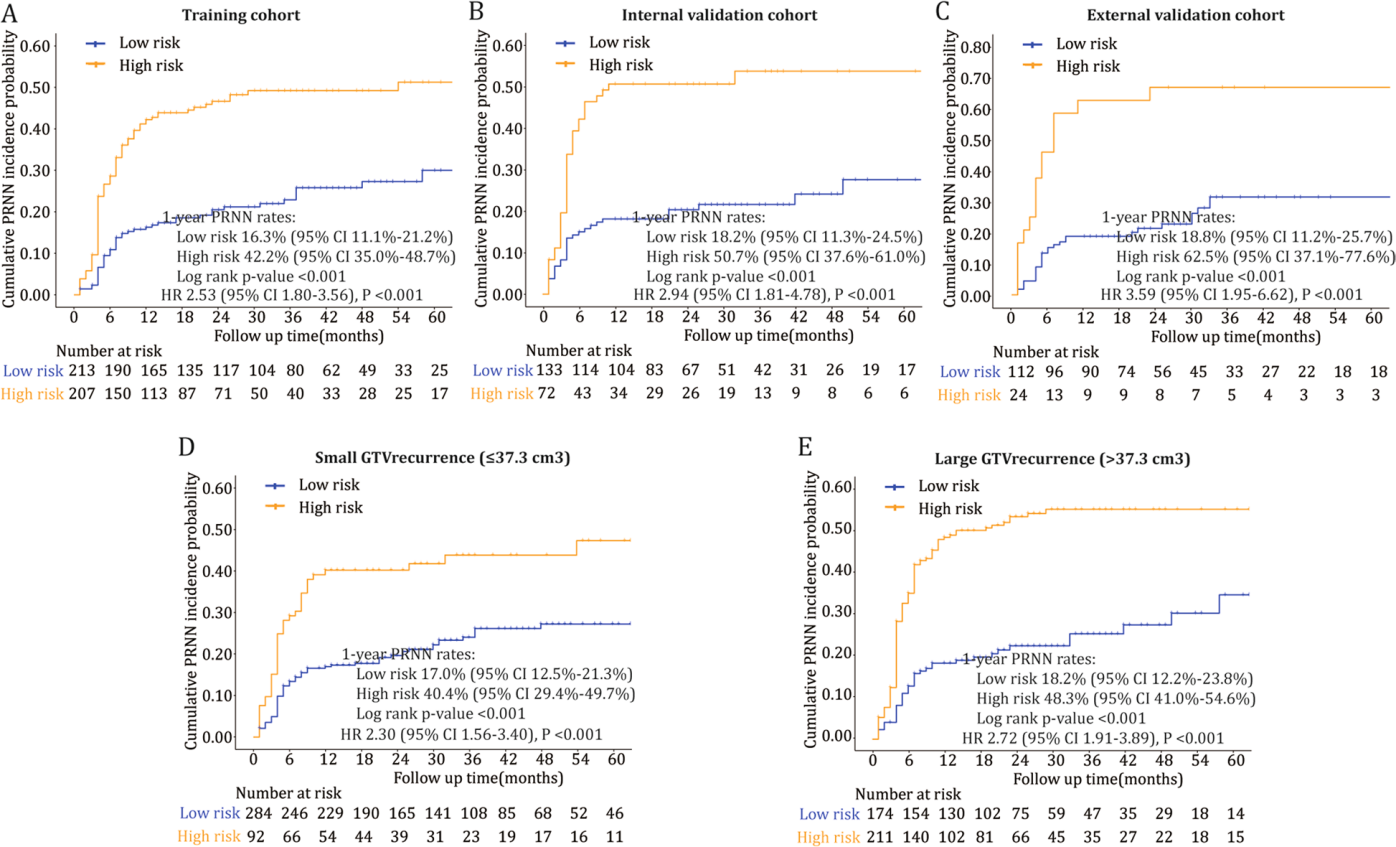
Risk stratification of post-radiation nasopharyngeal necrosis using the radiomic signature. **A–C** Cumulative incidences of post-radiation nasopharyngeal necrosis (PRNN) in the training (**A**), internal validation (**B**), and external validation (**C**) cohorts. **D–E** Cumulative PRNN incidences in patients with small (**D**) and large (**E**) tumors. Hazard ratios (HRs) were estimated using univariate Cox regression analyses. GTV gross tumor volume, CI confidence interval

With a cutoff value of 0.735, the signature exhibited good risk stratification of PRNN. The high-risk signature was associated with higher incidences of PRNN than the low-risk signature in all cohorts (1-year PRNN rates 42.2–62.5% vs. 16.3–18.8%, *P* < 0.001, Fig. 4A–C) and patients with small and large GTVs (*P* < 0.001, Fig. 4D–E). Multivariate Cox analyses indicated that the radiomic signature was an independent predictor of PRNN (hazard ratio (HR) 2.41, 95% CI 1.68–3.44, *P* < 0.001) after adjustment for sex, age, DFI, GTV_recurrence_, and re-irradiation dose (Additional file 1: Table S10). The signature could predict PRNN risk at different timepoints after re-radiotherapy (nomogram, Additional file 1: Figure S7).

### Survival outcomes associated with the signature

Patients with high-risk signature were at a higher risk of death from grade 5 PRNN-related factors and all causes than those with low-risk signature (Additional file 1: Figure S8). Multivariate analyses revealed that the signature was an independent predictor of G5-NFS and OS adjusted for sex, age, DFI, GTV_recurrence_, and re-irradiation dose (Additional file 1: Table S10), confirming its significant prognostic value.

### Biological processes associated with the signature

**Fig. 5:**
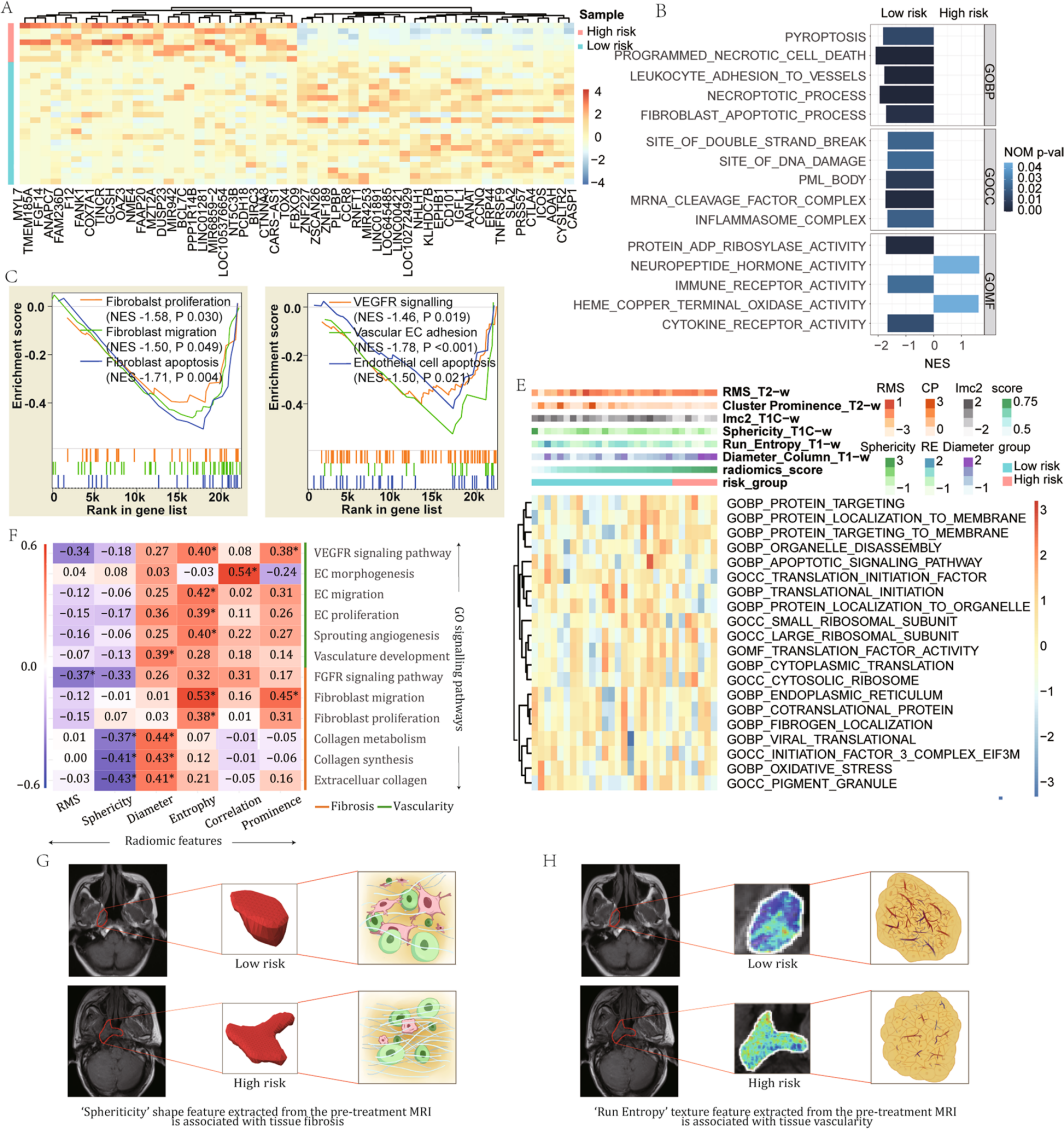
Biological processes associated with the radiomic signature for predicting post-radiation nasopharyngeal necrosis. **A** Expression of the top 50 positively and negatively ranked genes for the radiomic signature based on 29 cases (7 high-risk vs 22 low-risk patients) recruited from the Sun Yat-sen University Cancer Centre. The genes were clustered based on the correlation distance metric. **B** Significantly enriched gene sets from the Gene ontology (GO) collection using ClusterProfiler of the ranked genes. **C** GO-based fibroblast associated processes enriched with the radiomics signature. **D** GO-based vascularity associated processes enriched with the radiomics signature. **E** Heatmap of the GO-based single-sample enrichment scores in individual samples. **F** Spearman rank correlation coefficient matrix of the GO-based single-sample enrichment scores and the individual radiomic features included in the signature. *Statistical significance (*P* < 0.05). **G** Visualization of association between the shape feature (Sphericity) extracted from pre-treatment magnetic resonance imaging (MRI) and tissue fibrosis. **H** Visualization of association between the texture feature (Run Entropy) extracted from pre-treatment MRI and tissue vascularity. NES normalized enrichment score, EC endothelial cell, VEGFR vascular endothelial growth factor receptor, FGFR fibroblast growth factor receptor

Of the 29 included patients, 7 were classified as high-risk and the remaining 22 patients as low-risk. A total of 22,291 annotated genes were ranked by their association with the radiomic signature (Fig. 5A). GSEA indicated that the biological processes involved in fibroblast function and vascularity were significantly associated with the radiomic signature (Fig. 5B-D, Additional file 1: Table S11).

The ssGSEA scores of GO-based biological processes were calculated for individual patients and their associations with the six radiomic features were analyzed (Fig. 5E and F). The root mean squared feature was correlated with fibroblast growth factor receptor signaling pathway. Sphericity and diameter were associated with extracellular components indicative of tissue fibrosis (Fig. 5F and G); diameter also correlated with vasculature development. Three texture features were associated with fibroblast function, endothelial cell function, angiogenesis, and vascular endothelial growth factor receptor signaling pathway (Fig. 5F and H).

## Discussion

Consistent with previous studies [7, 8], we observed 34% of patients developed PRNN after curative re-radiotherapy, and grade 5 PRNN accounted for 24% of death for all the patients and over 50% of death for patients with PRNN (Fig. 1 and Additional file 1: Figure S4). With a large sample of re-irradiated patients in NPC endemic area, we found that the radiomics profile of pre-treatment MR images could reveal the nasopharyngeal tolerance to re-radiotherapy and predict PRNN development. The radiomic signature was generalized to different centers, MRI parameters, and patient subgroups with distinct demographics, tumor stage, and treatment settings. It was a radio-biomarker of radiation-injury associated processes regarding fibrosis and vascularity and could be incorporated into routine clinical practice at minimal cost and invasion. Such a tool would be useful to guide treatment of re-radiotherapy for patients with LRNPC.

Radiomics has been widely applied in precision radiation oncology [25]. This study was the first attempt to investigate the role of radiomics in the prediction of PRNN, which is a severe radiation-related complication. We assumed that the radiomics method could detect encrypted information in the recurrent lesion regarding residual damage from prior radiotherapy and heterogenous repair ability, which is critical for PRNN risk assessment. Notably, the proposed radiomic signature showed discriminative power (AUC 0.713–0.756). The high-risk signature was associated with remarkably higher risks of PRNN and associated deaths than the low-risk signature. These findings justify the clinical utility of the radiomic signature to guide re-radiotherapy. High-risk patients should be counselled about suboptimal survival if treated with curative re-radiotherapy because of the high incidence of PRNN (42.2–62.5%) and the associated adverse outcomes. Palliative radiation at lower doses or even the omission of re-radiotherapy should be considered for these individuals [26]. Other palliative treatments including chemotherapy, immunotherapy, and targeted therapy could be applied [3, 27, 28]. Conversely, low-risk patients may be candidates for re-radiotherapy. However, considering probability of PRNN in this group (16.3–18.8%) and risks of other radiation-induced toxicities, comprehensive pre-treatment evaluation of the patient status and close surveillance of PRNN signs are still needed for low-risk patients.

GTV_recurrence_ was a strong predictor of PRNN [7, 12]. However, even for patients with small GTV_recurrence_ (≤ 37.3 cm3), the incidence of PRNN was still high (22.6%). The radiomic signature was able to stratify risks of PRNN within patient subgroups with small and large GTV_recurrence_ (Fig. 4D–E). Adding this volume factor to the signature did not increase its predictive value. This is predominantly because some radiomic features (e.g., maximum 2D diameter column) are closely related with tumor volume. Moreover, the radiomic signature is considered as a better predictor of PRNN than pure volume factor, as it detects more information concerning tissue physiopathology. The re-irradiation dose is another important dosimetric factor [7, 8]. Theoretically, the re-irradiation dose could provide additional treatment information complementary to the pre-treatment MRI data; however, it failed to significantly improve our model. Most of the patients in this study were treated with re-irradiation doses of 60–64 Gy, and < 7% of patients received doses of ≥ 68 Gy, which were distinct from those reported previously [7, 8]. Within this limited range, the re-irradiation dose had little predictive value.

In radiogenomics analysis, pathways involved in fibroblast function and vascularity were found associated with the PRNN radiomic signature. Fibrosis and reduced vascularity are two dominant features of radiation-induced tissue damage. Tissue fibrosis is a late phase of radiation-induced fibroatrophy; the molecular mechanisms involved in this process are fibroblast dysfunction and extracellular matrix deposition [29]. After prior high doses of irradiation, the nasopharyngeal tissue experiences various degrees of tissue fibrosis, which constrains the local blood supply and repair ability, thus determining the tolerance of the nasopharynx to re-irradiation. Further, reduced vascularity, another sequela of prior radiation, may lead to hypoxia and cell death [30, 31], and is involved in the PRNN development.

Several limitations should be noted. First, this is a retrospective study, and the included patients were all from China. Further prospective studies in endemic and non-endemic areas are needed. Future studies can incorporate multicenter cohorts into the model training and tuning, so that the model can be more accurate and generalized. Second, some clinical features such as prior radiation-induced toxicities were not reported, owing to difficulties in accessing the data in this retrospective study. Third, not all the patients included in this study had pathological examinations to exclude cancer recurrences when diagnosing PRNN. There exist difficulties to obtain tissue from the site where PRNN occur, especially for patients with severe and emergent situations. Fourth, the transcriptomics data were used to correlate radiomics signature with the underlying biological processes, but not incorporated in the construction of predictive model because of limited sample size. Combining high throughput sequencing data from transcriptomics, genomics, and proteomics with the current radiomics model may further improve the predictive ability [32]. Currently some advanced technologies can dissect the biological activities at a single cell level and including spatial information [33, 34]. The incorporation of these sequencing technologies with the radiomics model has great potential and deserves investigation in future studies.

## Conclusions

Recurrent tumors are heterogenous with different cellular and molecular functions. Biological heterogeneity contributes to adverse clinical outcomes including intolerance to radical treatment and decreased survival. In this study, multiscale data from MR images and transcriptomics were used to construct a radiogenomic signature to predict PRNN following re-radiotherapy in patients with LRNPC. The proposed signature has prognostic value and serves as a noninvasive clinical tool to guide re-radiotherapy.

### Supplementary Information

**Additional file 1: Supplementary methods A1-A13.** **Figures S1-S8.** **Tables S1-S11.** **Methods A1.** Inclusion and exclusion criteria. **Methods A2.** Treatment protocols. **Methods A3.** Patient characteristics. **Methods A4.** Diagnosis of PRNN and follow-up protocol. **Methods A5.** ROI segmentation. **Methods A6.** Radiomic features extraction. **Methods A7.** Feature selection and radiomic signature building. **Methods A8.** Candidate clinical variables and model building. **Methods A9.** Model performance. **Methods A10.** Transcriptome sequencing. **Methods A11.** GSEA. **Methods A12.** Radiomics quality score. **Methods A13.** Random forest model parameters and the included radiomic features. **Figure S1.** Clinical manifestations of post-radiation nasopharyngeal necrosis. **Figure S2.** The diagnostic workflow of post-radiation nasopharyngeal necrosis. **Figure S3.** Segmentation criteria of region-of-interest. **Figure S4.** Overall survival of patients with and without post-radiation nasopharyngeal necrosis. **Figure S5.** Pair-wise correlations among six selected radiomic features. **Figure S6.** Confusion matrix for the prediction of PRNN using radiomics model. **Figure S7.** Nomogram predicting risk of post-radiation nasopharyngeal necrosis at different timepoints. **Figure S8.** Prognostic significance of the radiomic signature. **Table S1.** Acquisition parameters of MRI scanning. **Table S2.** Summary of clinical outcomes of patients in training and validation cohorts. **Table S3.** Choices of methods for radiomic signature development. **Table S4.** Number of features remained after each step of feature selection. **Table S5.** Details of the six radiomic features included in the random forest model. **Table S6.** Candidate clinical and radiomics variables for prediction of PRNN in training cohort. **Table S7.** Performance of the radiomics and clinical models in training and validation cohorts. **Table S8.** Generalizability of the radiomic signature across medical centers and MRI parameters. **Table S9.** Generalizability of the radiomic signature across patient subgroups. **Table S10.** Prognostic significance of the radiomic signature in multivariate Cox analyses. **Table S11.** Core enrichment genes of the biological processes associated with the radiomic signature.

## Data Availability

The code is available at https://github.com/CarnoZhao/NPC_PRNN. The Original images and representative ROIs are available from the corresponding authors on reasonable request.

## Abbreviations

AUC: Area under the curve
CI: Confidence intervals
DFI: Disease-free interval
G5-NFS: Grade 5 necrosis-free survival
GLCM: Gray Level Co-occurrence Matrix
GLDM: Gray Level Dependence Matrix
GLRLM: Gray Level Run-Length Matrix
GLSZM: Gray Level Size Zone Matrix
GO: Gene Ontology
GSEA: Gene set enrichment analysis
GTV: Gross tumor volume
ICC: Intra/inter-class correlation coefficients
IMRT: Intensity modulated radiotherapy
LRNPC: Locally recurrent nasopharyngeal carcinoma
MRI: Magnetic resonance imaging
NPC: Nasopharyngeal carcinoma
OS: Overall survival
PRNN: Post-radiation nasopharyngeal necrosis
RFE: Recursive feature elimination
ROC: Receiver operating characteristic
ROI: Region-of-interest
ssGSEA: Single-sample gene set enrichment analysis
SVM: Linear support vector machines
SYSUCC: Sun Yat-sen University Cancer Center
