# Supplementary material for: Radiomic signatures reveal multiscale intratumor heterogeneity associated with tissue tolerance and survival in re-irradiated nasopharyngeal carcinoma: a multicenter study
Source: BMC Med. 2023 Nov 27;21:464. doi: 10.1186/s12916-023-03164-3 (PMC10683300; doi:10.1186/s12916-023-03164-3)
Supplement: Supplementary file 1 — Additional file 1: Supplementary methods A1-A13. Figures S1-S8. Tables S1-S11. Methods A1. Inclusion and exclusion criteria. Methods A2. Treatment protocols. Methods A3. Patient characteristics. Methods A4. Diagnosis of PRNN and follow-up protocol. Methods A5. ROI segmentation. Methods A6. Radiomic features extraction. Methods A7. Feature selection and radiomic signature building. Methods A8. Candidate clinical variables and model building. Methods A9. Model performance. Methods A10. Transcriptome sequencing. Methods A11. GSEA. Methods A12. Radiomics quality score. Methods A13. Random forest model parameters and the included radiomic features. Figure S1. Clinical manifestations of post-radiation nasopharyngeal necrosis. Figure S2. The diagnostic workflow of post-radiation nasopharyngeal necrosis. Figure S3. Segmentation criteria of region-of-interest. Figure S4. Overall survival of patients with and without post-radiation nasopharyngeal necrosis. Figure S5. Pair-wise correlations among six selected radiomic features. Figure S6. Confusion matrix for the prediction of PRNN using radiomics model. Figure S7. Nomogram predicting risk of post-radiation nasopharyngeal necrosis at different timepoints. Figure S8. Prognostic significance of the radiomic signature. Table S1. Acquisition parameters of MRI scanning. Table S2. Summary of clinical outcomes of patients in training and validation cohorts. Table S3. Choices of methods for radiomic signature development. Table S4. Number of features remained after each step of feature selection. Table S5. Details of the six radiomic features included in the random forest model. Table S6. Candidate clinical and radiomics variables for prediction of PRNN in training cohort. Table S7. Performance of the radiomics and clinical models in training and validation cohorts. Table S8. Generalizability of the radiomic signature across medical centers and MRI parameters. Table S9. Generalizability of the radiomic signature across patient subgrou [file 12916_2023_3164_MOESM1_ESM.pdf]

Supplementary appendix

Radiomic signatures reveal multiscale intratumor heterogeneity associated with tissue tolerance and survival in re-irradiated nasopharyngeal carcinoma: a multicentre study

Appendix

Table of contents

Supplementary methods 2
Supplementary A1. Inclusion and exclusion criteria 2
Supplementary A2. Treatment protocols 2
Supplementary A3. Patient characteristics 2
Supplementary A4. Diagnosis of PRNN and follow-up protocol 3
Supplementary A5. ROI segmentation 3
Supplementary A6. Radiomic features extraction 3
Supplementary A7. Feature selection and radiomic signature building 3
Supplementary A8. Candidate clinical variables and model building 4
Supplementary A9. Model performance 4
Supplementary A10. Transcriptome sequencing 4
Supplementary A11. GSEA 5
Supplementary A12. Radiomics quality score 5
Supplementary A13. Random forest model parameters and the included radiomic features 6
References 7
Figure S1. Clinical manifestations of post-radiation nasopharyngeal necrosis. 8
Figure S2. The diagnostic workflow of post-radiation nasopharyngeal necrosis. 10
Figure S3. Segmentation criteria of region-of-interest. 11
Figure S4. Overall survival of patients with and without post-radiation nasopharyngeal necrosis. 12
Figure S5. Pair-wise correlations among six selected radiomic features. 13
Figure S6. Confusion matrix for the prediction of PRNN using radiomics model. 14
Figure S7. Nomogram predicting risk of post-radiation nasopharyngeal necrosis at different timepoints. 15
Figure S8. Prognostic significance of the radiomic signature. 16
Table S1. Acquisition parameters of MRI scanning. 17
Table S2. Summary of clinical outcomes of patients in training and validation cohorts. 18
Table S3. Choices of methods for radiomic signature development. 19
Table S4. Number of features remained after each step of feature selection. 20
Table S5. Details of the six radiomic features included in the random forest model. 21
Table S6. Candidate clinical and radiomics variables for prediction of PRNN in training cohort. 22
Table S7. Performance of the radiomics and clinical models in training and validation cohorts. 23
Table S8. Generalizability of the radiomic signature across medical centers and MRI parameters. 24
Table S9. Generalizability of the radiomic signature across patient subgroups. 25
Table S10. Prognostic significance of the radiomic signature in multivariate Cox analyses. 26
Table S11. Core enrichment genes of the biological processes associated with the radiomic signature. 27

Supplementary methods

Supplementary A1. Inclusion and exclusion criteria

The inclusion criteria were as follows: pathologically confirmed LRNPC, no evidence of distant metastasis at recurrence, disease-free interval (DFI, defined as the interval between the end of the first course of radiotherapy and the diagnosis of recurrence) of >6 months, treated with the second course of definitive radiotherapy (≥60 Gy), and available pre-treatment MRI. The exclusion criteria included having a history of other malignancies, prior surgery to nasopharynx, presence of nasopharyngeal necrosis before re-radiotherapy and loss to follow up. The radiogenomics study selected patients with additional inclusion criteria as follows: had frozen tissue samples deposited in the SYSUCC specimen library; samples with enough RNA total amount and good RNA integrity for subsequent sequencing.

Supplementary A2. Treatment protocols

All medical centres used a similar re-radiotherapy planning protocol as previously described[1,2]. Patients were treated with a second course of IMRT or helical tomotherapy. Prescribed doses for $GTV_{recurrence}$ were 60.0-70.0 Gy in 27-35 fractions (conventional fraction) or 50-54 fractions (hyperfraction) at the Sun Yat-sen University Cancer Center (SYSUCC), 60.0-70.0 Gy in 28-33 fractions (conventional fraction) or 50 fractions (hyperfraction) at the Cancer Hospital Chinese Academy of Medical Sciences (CHCAMS), 60.0-70.0 Gy in 30-35 fractions at the Fudan University Shanghai Cancer Center (FUSCC), and 60.0-70.0 Gy in 25-35 fractions at the Nanfang Hospital of Southern Medical University (NHSMU). Dose constraints to the critical organs were determined by the standard threshold doses, DFI, and patient's performance status. The target volumes were delineated based on fused MRI and computed tomography simulation images at a slice thickness of 3 mm. A thermoplastic shell was applied for immobilization. The gross tumor volume (GTV) included all the contrast-enhanced lesions and lymph nodes. The clinical target volumes (CTV) were designed to encompass microscopic disease including the GTV plus a 5-mm to 10-mm margin and a smaller margin (<3 mm) allowed near critical intracranial structures or the spinal cord. The CTV also included the entire nasopharynx and the lymph node positive involved regions. No elective reirradiation of uninvolved regional lymph nodes was performed. Critical normal structures, including the brainstem, spinal cord, parotid glands, optic nerves, chiasm, lens, eyeballs, temporal lobes, temporomandibular joints, mandible, and hypophysis were contoured and set as organs at risk (OARs) during optimization. Additional 2-mm to 3-mm margins were added to the CTV and GTV to create the planning target volume (PTV) for setup variability and internal motion. Cisplatin-based induction chemotherapy (cisplatin plus 5-fluorouracil, paclitaxel or gemcitabine) and/or concurrent chemotherapy (intravenous cisplatin 80-100 mg/m2 every 3 weeks) was administrated at the physician's discretion, given that the role of chemotherapy in re-irradiated patients remains unclear[3].

Supplementary A3. Patient characteristics

Patient demographics, tumor stage and treatment parameters were collected, including age, sex, body mass index (BMI), complications of hypertension and diabetes, DFI, recurrent tumor stage, pathological type, baseline hemoglobin level, administration of concurrent chemotherapy and re-radiotherapy planning indices including radiation dose, fractionation, and $GTV_{recurrence}$. Because of the variation in the dose-fraction size of re-radiotherapy, all re-irradiation doses were converted to equivalent doses in 2-Gy fraction values using the following equation: total dose * (dose per fraction + 10) / (2 + 10), where 10 = a/b ratio for head and neck cancer[2]. All patients were restaged according to the 8th American Joint Committee on Cancer Union for International Cancer Control staging system. Radiotherapy-induced toxicities were recorded and graded according to the toxicity criteria of the Radiation Therapy Oncology Group.

Supplementary A4. Diagnosis of PRNN and follow-up protocol

PRNN was diagnosed according to clinical symptoms, nasopharyngeal endoscopy, MRI, and pathological examinations (Figure S1)[4,5]. The typical endoscopic manifestations of PRNN included a denaturalized nasopharyngeal mucosa, necrotic substances coverage, and an exposed skull base. The MRI findings of PRNN included a discontinuous nasopharyngeal mucosa line, soft tissue defects without enhancement, and skull base osteonecrosis. For lesions that make differentiating PRNN and tumor relapse difficult, endoscopic biopsies or surgery were performed for pathological confirmation. Patients who relapsed prior to or concurrent with PRNN were excluded for diagnosis of PRNN. The diagnostic workflow of PRNN was shown in supplementary Figure S2. PRNN was diagnosed by professional radiation oncologists at the four medical centres. All patients were followed up according to the routine practice of the study centres for ≥12 months after re-radiotherapy or until death. Patients were evaluated every 3 months during the first year after the completion of treatment, every 6 months during the subsequent 2 years and every year thereafter. Nasopharyngeal endoscopy, MRI of the head and neck, chest radiography, abdominal sonography, and plasma Epstein Barr virus DNA measurement were routinely performed.

Supplementary A5. ROI segmentation

The recurrent tumor localized in the nasopharynx was selected as the main region-of-interest (ROI_nx) to predict PRNN because it is within the high dose zones of the two courses of radiotherapy and the area in which PRNN events occurred (Figure S1). The whole tumor including the lesions beyond the nasopharynx and invading the skull base and intracranial cavity was considered as a second ROI (ROI_total) to explore the potential predictive efficacy of the model. ROI segmentation was performed on three axial MRI sequences by professional radiation oncologists in the four medical centres (Sun R, Ou XM, Zhang Y, and Guan J) using the ITK-SNAP software (version 3.8.0; www.itksnap.org). Each segmentation was validated by an experienced radiologist (Lv XF) and senior radiation oncologists in the four medical centres (Chen QY, Hu CS, Yi JL, and Wu DH). We used different labels to differentiate the tumor lesion localized in the nasopharynx (label 1) from those invading the skull base and intracranial cavity (label 2, Figure S3). After 4 weeks, 50 patients in the training cohort were randomly selected and their ROIs were segmented again by the same oncologist (Sun R) and another professional radiation oncologist (Luo DH), to assess the intra-reader and inter-reader reproducibility of radiomic features.

Supplementary A6. Radiomic features extraction

The extracted features consisted of three types: first order statistics, shape, and texture (Gray Level Co-occurrence Matrix [GLCM], Gray Level Dependence Matrix [GLDM], Gray Level Run-Length Matrix [GLRLM], and Gray Level Size Zone Matrix [GLSZM])[6]. After applying three-dimensional wavelet or Laplacian of Gaussian (LoG) transformations to the original images, the features were recalculated and included. Wavelet transformation decomposes the data into high and low-frequency components, to detect the hidden temporal features and improve the signal-to-noise ratio of images. LoG is an edge enhancement filter with different sigma levels (1 mm, 3 mm, and 5 mm) indicating the coarseness of textures. Note that, all the radiomic features conform to Image Biomarker Standardization Initiative (IBSI)[8]. A total of 2106 features (702 features in each MRI series) were extracted for ROI analysis.

Supplementary A7. Feature selection and radiomic signature building

To avoid model overfitting and improve performance, feature selection was performed in the training

cohort as follows: first, we used intra/inter-class correlation coefficients (ICCs) to assess the reproducibility of extracted radiomic features based on re-segmented data. Only stable features with ICCs of >0.7 were selected. Second, each feature was weighed on its value in discriminating the 1-year PRNN from the non-PRNN cases using three feature ranking methods; these methods included univariate logistic regression, lasso regression and linear support vector machines (SVM) binary classifier. Only the 100 most important features in each series were used to construct a compact candidate feature list for further analysis. Third, a fine selection approach was applied to select the most representative features from the compact candidate feature list and generate candidate classification models. We applied a grid search strategy to determine the best number of features to be remained, which is denoted by K. We filtered the selected features recursively using a recursive feature elimination (RFE) approach based on the model's accuracy to predict PRNN on repeated five-fold cross validation and retained K features per MR series, for 3K features in total. To address the multi-dimensionality and possible redundancy of the radiomic features, we limited the correlation coefficient between features to <0.7 in RFE to retain the uncorrelated features. In this study, three widely-used modelling algorithms were used to generate models, e.g., multivariate logistic regression, linear SVM, and random forest. All three modelling algorithms were performed on the three compact candidate feature lists attained in the feature ranking step and two sets of ROIs to generate a total of 3×3×2 candidate models. Finally, the model that achieved the highest performance in the training cohort was selected.

Supplementary A8. Candidate clinical variables and model building

Candidate clinical predictor variables for PRNN included age, sex, BMI, DFI, recurrent T category, $GTV_{recurrence}$, re-irradiation dose, accumulated GTV dose, combination with chemotherapy, and baseline hemoglobin level; these factors were carefully selected according to their clinical significance and possible association with PRNN as shown in previous studies. Each predictor variable was assessed with its value in discriminating the 1-year PRNN from the non-PRNN cases and categorized into a binary variable to perform subgroup analyses (Table S6). Finally, four clinical factors that had the best discriminative ability in the training cohort (including sex, age, DFI, and $GTV_{recurrence}$, AUC >0.5), along with the important dosimetric factor of re-irradiation dose were used to construct the clinical model, using the optimal modelling algorithm among multivariate Logistic regression, SVM, and Random Forest.

Supplementary A9. Model performance

The performance of the radiomic signature was comprehensively assessed as follows: first, ROC curves were used to assess the prediction accuracy of the radiomic signature. Second, stratification analyses according to different clinical factors and MRI acquisition parameters were performed to assess the generalizability of the model. Third, a calibration curve was plotted to assess the calibration of the model. Fourth, decision curve analysis was conducted to evaluate the clinical utility of the model in guiding re-radiotherapy by quantifying the net benefits. Fifth, a nomogram was designed to predict PRNN in a continuous fashion depending on the value of the radiomic signature[7]. Sixth, subgroup analyses were performed based on the cutoff value in order to assess the prognostic value of the radiomic signature for (i) PRNN; (ii) G5-NFS; and (iii) OS in univariate Kaplan-Meier curves and multivariate Cox proportional hazard models adjusted for sex, age, DFI, $GTV_{recurrence}$, and re-irradiation dose.

Supplementary A10. Transcriptome sequencing

Tissue samples obtained within four weeks of pre-treatment MRI were retrieved for the 29 patients included in the radiogenomics study (SYSUCC-C) to perform standard transcriptome next-generation

sequencing. Total RNA was extracted using a mirVana miRNA Isolation Kit (Ambion) following the manufacturer's protocol. RNA integrity was evaluated using the Agilent 2100 Bioanalyzer (Agilent Technologies, Santa Clara, CA, USA). Samples with an RNA Integrity Number (RIN) of ≥7 were subjected to the subsequent analysis. The RNA sequencing libraries were constructed using TruSeq Stranded Total RNA with Ribo-Zero Gold and then sequenced on the Illumina sequencing platform (HiSeqTM 2500). The 150-bp paired-end reads from RNA-seq were mapped to the human reference genome (UCSC hg38) using STAR v020201. RSEM v1.3.0 was then used to perform gene expression quantification.

Supplementary A11. GSEA

Using the signal-to-noise measure in GSEA, we ranked 22,291 annotated genes by their association with the PRNN radiomic signature (high-risk group vs. low-risk group). The predefined gene sets for the GO based biological processes were acquired from the Molecular Signatures Database (MSigDB v7.4) platform and used to perform pre-ranked GSEA with the appropriate software (GSEA 4.0.3, https://www.gsea-msigdb.org). To gain further insight into the biological implications of the individual radiomic features selected to build the signature, we performed ssGSEA using the Gene Set Variation Analysis (GSVA) package of R. For individual patients, an enrichment score was calculated for each predefined gene set in GO-based biological processes. The association of the six radiomic features used to build the PRNN radiomic signature with the GO based biological processes were analyzed using Spearman's rank correlation coefficient.

Supplementary A12. Radiomics quality score

The radiomics quality score of the dataset was high and estimated at 66.67% (24 out of 36 points):
- Image protocol quality - well-documented image protocols (for example, contrast, slice thickness, energy, etc.) and/or usage of public image protocols allow reproducibility/replicability. 1/1 point
- Multiple segmentations - possible actions are: segmentation by different physicians/algorithms/software, perturbing segmentations by (random) noise, segmentation at different breathing cycles. Analyse feature robustness to segmentation variabilities. 1/1 point
- Phantom study on all scanners - detect inter-scanner differences and vendor-dependent features. Analyse feature robustness to these sources of variability. 1/1 point
- Imaging at multiple time points - collect images of individuals at additional time points. Analyse feature robustness to temporal variabilities (for example, organ movement, organ expansion/ shrinkage). 0/1 point
- Feature reduction or adjustment for multiple testing - decreases the risk of overfitting. Overfitting is inevitable if the number of features exceeds the number of samples. Consider feature robustness when selecting features. 3/3 points
- Multivariable analysis with non-radiomics features (for example, EGFR mutation) - is expected to provide a more holistic model. Permits correlating/inferencing between radiomics and non-radiomics features. 1/1 point
- Detect and discuss biological correlates - demonstration of phenotypic differences (possibly associated with underlying gene–protein expression patterns) deepens understanding of radiomics and biology. 1/1 point
- Cut-off analyses - determine risk groups by either the median, a previously published cut-off or report a continuous risk variable. Reduces the risk of reporting overly optimistic results. 1/1 point
- Discrimination statistics - report discrimination statistics (for example, C-statistic, ROC curve, AUC) and their statistical significance (for example, p-values, confidence intervals). One can also apply resampling method (for example, bootstrapping, cross-validation). 2/2 points

- Calibration statistics - report calibration statistics (for example, Calibration-in-the-large/slope, calibration plots) and their statistical significance (for example, P-values, confidence intervals). One can also apply resampling method (for example, bootstrapping, cross-validation). 2/2 points
- Prospective study registered in a trial database - provides the highest level of evidence supporting the clinical validity and usefulness of the radiomics biomarker. 0/7 points
- Validation - the validation is performed without retraining and without adaptation of the cut-off value, provides crucial information with regard to credible clinical performance. 5/5 points
- Comparison to 'gold standard' - assess the extent to which the model agrees with/is superior to the current 'gold standard' method. 2/2 points
- Potential clinical utility - report on the current and potential application of the model in a clinical setting (for example, decision curve analysis). 2/2 points
- Cost-effectiveness analysis - report on the cost-effectiveness of the clinical application (for example, QALYs generated) 0/1 point
- Open science facilitates knowledge transfer and reproducibility of the study. 2/4 point

Supplementary A13. Random forest model parameters and the included radiomic features

Within the training cohort, six radiomic features were selected to build the PRNN signature using the random forest model (Table S5). There are 500 trees in one forest model. The max-depth of each tree is 2. We random subsampled 75% from the total training samples to build each tree. Entropy criterion and balanced class weights were also used in forest building.

Definitions of the radiomic features have been derived from the pyradiomics library (https://pyradiomics.readthedocs.io/en/latest/index.html, last accessed May 22, 2021) and summarized below.

(i) Root Mean Squared (RMS): first order statistic, the square-root of the mean of all the squared intensity values, measuring the magnitude of the attenuation values in the whole ROI, disregarding the spatial relationship of the individual voxels.

$$\textbf{RMS} = \sqrt{\frac{1}{N_p}\sum_{i=1}^{N_p}(\mathrm{X}(i)+c)^2}$$

where $\boldsymbol{X}$ is the ROI with $\boldsymbol{N_p}$ pixels, $\boldsymbol{c}$ a value that shifts the intensities to prevent negative values in $\boldsymbol{X}$. This ensures that voxels with the lowest gray values contribute the least to Energy, instead of voxels with gray level intensity closest to 0.

(ii) Sphericity: shape feature, measuring the roundness of the shape of ROI relative to a sphere. It is a dimensionless measure, independent of scale and orientation. The value range is 0<sphericity≤1, where a value of 1 indicates a perfect sphere (a sphere has the smallest possible surface area for a given volume, compared to other solids).

$$\textbf{Sphericity} = \frac{\sqrt[3]{36\pi V^2}}{A}$$

where $\boldsymbol{V}$ is the volume of ROI in mm3 and $\boldsymbol{A}$ the surface area of ROI in mm2.

(iii) Maximum 2D Diameter (Column): shape feature, the largest pairwise Euclidean distance between ROI surface voxels in the row-slice (usually the coronal) plane.

(iv) Run Entropy (RE) of Gray Level Run Length Matrix (GLRLM): texture feature, measuring the uncertainty/randomness in the distribution of run lengths and gray levels. A higher value indicates more heterogeneity in the texture patterns.

$$\textbf{RE} = -\sum_{i=1}^{N_g}\sum_{j=1}^{N_r} p(i,j|\theta)\log_2\left(p(i,j|\theta)+\epsilon\right)$$

where N_g is the number of discreet intensity values in the image, N_r the number of discreet run lengths in the image, ***P(i,j|θ)*** the run length matrix for an arbitrary direction θ, ***p(i,j|θ)*** the normalized run length matrix, defined as $p(i,j|\theta) = \frac{P(i,j|\theta)}{N_z(\theta)}$, and ϵ an arbitrarily small positive number (≈2.2×10−16).

(v) Informational Measure of Correlation 2 (IMC 2) of Gray Level Co-occurrence Matrix (GLCM): texture feature, measuring the correlation between the probability distributions of i and j (quantifying the complexity of the texture).

$$\textbf{IMC 2} = \sqrt{1 - e^{-2(HXY2-HXY)}}$$

(vi) Cluster Prominence (CP) of GLCM: texture feature, measuring the skewness and asymmetry of the texture. A higher value implies more asymmetry around the mean while a lower value indicates a peak near the mean value and less variation around the mean.

$$\textbf{CP} = \sum_{i=1}^{N_g}\sum_{j=1}^{N_g}(i+j-\mu_x-\mu_y)^4 p(i,j)$$

where ***P(i, j)*** is the ***(i, j)***th entry in the GLCM, N_g the number of intensity levels in the image, μ_x the mean gray level intensity of P_x, μ_y the mean gray level intensity of P_y.

References

1. Tian YM, Zhao C, Guo Y et al. Effect of total dose and fraction size on survival of patients with locally recurrent nasopharyngeal carcinoma treated with intensity-modulated radiotherapy: a phase 2, single-center, randomized controlled trial. *Cancer-Am Cancer Soc*. 2014;120(22):3502-9
2. Li YQ, Tian YM, Tan SH et al. Prognostic Model for Stratification of Radioresistant Nasopharynx Carcinoma to Curative Salvage Radiotherapy. *J Clin Oncol*. 2018;36(9):891-899
3. Lee AWM, Ng WT, Chan JYW et al. Management of locally recurrent nasopharyngeal carcinoma. *Cancer Treat Rev*. 2019;79:101890-101890
4. Hua YJ, Chen MY, Qian CN et al. Postradiation nasopharyngeal necrosis in the patients with nasopharyngeal carcinoma. *Head Neck*. 2009;31(6):807-12
5. Yang Q, Zou X, You R et al. Proposal for a new risk classification system for nasopharyngeal carcinoma patients with post-radiation nasopharyngeal necrosis. *Oral Oncol*. 2017;67:83-88
6. van Griethuysen J, Fedorov A, Parmar C et al. Computational Radiomics System to Decode the Radiographic Phenotype. *Cancer Res*. 2017;77(21):e104-e107
7. Piessevaux H, Buyse M, Schlichting M et al. Use of early tumor shrinkage to predict long-term outcome in metastatic colorectal cancer treated with cetuximab. *J Clin Oncol*. 2013;31(30):3764-75
8. Zwanenburg A, Vallières M, Abdalah M A, et al. The image biomarker standardization initiative: standardized quantitative radiomics for high-throughput image-based phenotyping. Radiology, 2020, 295(2): 328-338

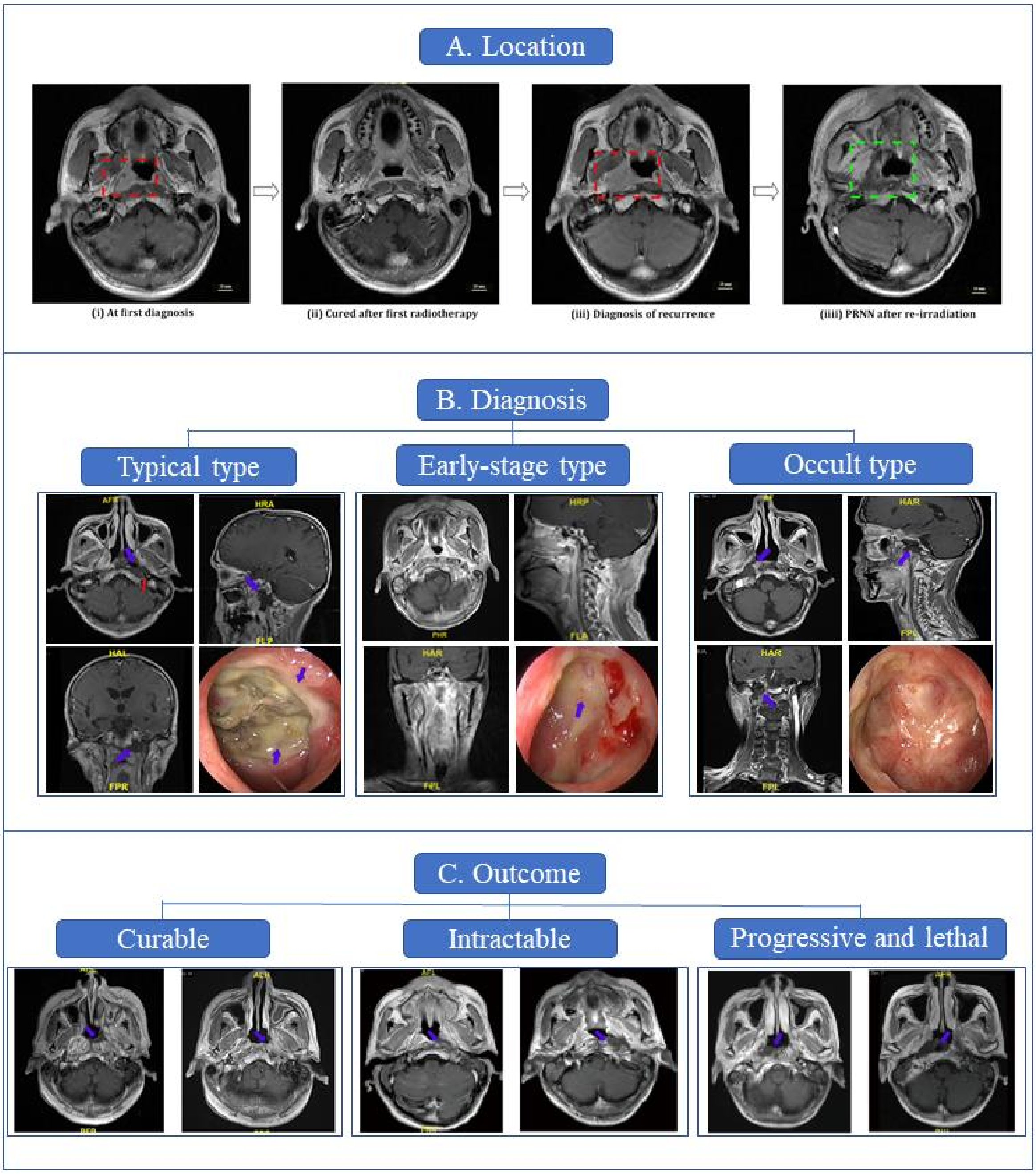

Figure S1. Clinical manifestations of post-radiation nasopharyngeal necrosis.

(A) Location of PRNN: usually in the recurrent tumor region and within the high dose zone of first radiotherapy. The four MRI pictures showed the same patient experiencing (i) first diagnosis of NPC; (ii) cure without PRNN after first definitive radiotherapy; (iii) diagnosis of recurrence at the same site two years after first definitive radiotherapy; (iiii) occurrence of PRNN after re-radiotherapy to the recurrent tumor region. (B) Diagnosis of PRNN faced with different scenarios in clinical practice: typical type, with apparent manifestations on both MRI and endoscope; early-stage type, with denaturalized mucosa under endoscope but no tissue defects on MRI; occult type, lesion occurred in the para-pharynx region was not seen under endoscope. Therefore, MRI and endoscope are two complementary methods for diagnosis of PRNN. (C) Outcome of PRNN: occasionally curable (patient 1), mostly intractable (patient 2) and rapid progressive (patient 3). Patient 1 had early-stage mucosa necrosis after re-radiotherapy (left) and was treated with conservative therapeutics including repeated

endoscopic debridement and antibiotics; three months later, the patient recovered from PRNN (right). Patient 2 had PRNN after re-radiotherapy (left) and was treated with conservative therapeutics; eight months later, the patient had no recovery from PRNN (right). Patient 3 also had PRNN after re-radiotherapy (left) and was treated with conservative therapeutics; in three months, the patient experienced rapid progression of PRNN (right) and soon died of massive bleeding. PRNN, post-radiation nasopharyngeal necrosis; MRI, Magnetic Resonance Imaging; NPC, nasopharyngeal carcinoma.

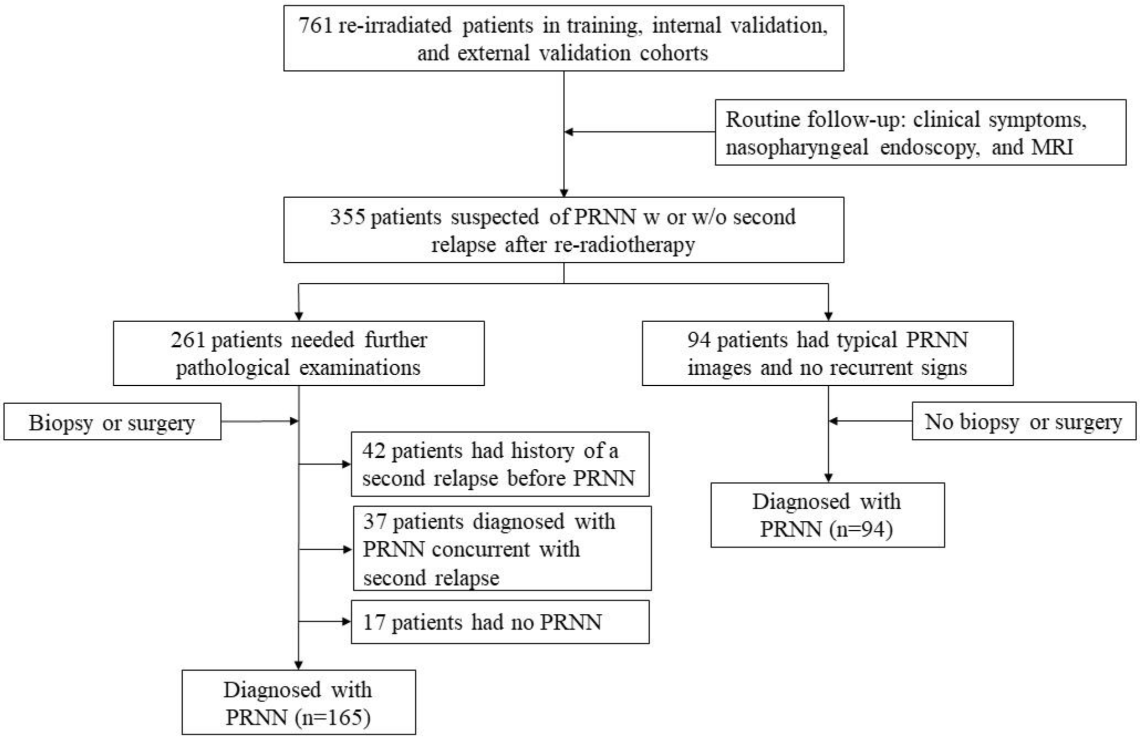

Figure S2. The diagnostic workflow of post-radiation nasopharyngeal necrosis.

A total of 761 patients with LRNPC who received curative re-radiotherapy were recruited from four medical centres and divided into training, internal validation, and external validation cohorts. All patients were followed up for clinical outcomes including PRNN, second relapse, and death after re-radiotherapy; outpatient visit, nasopharyngeal endoscopy and MRI of the head and neck were routinely performed. Overall, 355 patients were detected that suspected of PRNN during follow-up, some of whom also had a history of relapse. Among them, 94 patients who had typical PRNN imaging manifestations and no recurrent signs were diagnosed clinically as PRNN without biopsy. The other 261 patients underwent biopsy or surgery for diagnosis and treatment of PRNN; among them, 42 patients had history of relapse before PRNN, 37 patients had relapse concurrent with PRNN, and 17 patients had no evidence of PRNN, all of whom were excluded for diagnosis of PRNN. Finally, 165 patients diagnosed of PRNN after pathological confirmation.

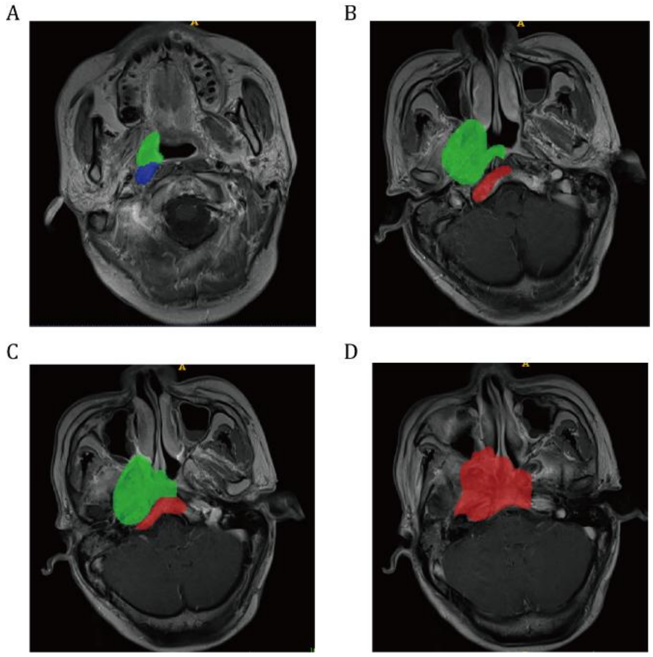

Figure S3. Segmentation criteria of region-of-interest.

Considering the complexities of structures that nasopharyngeal carcinoma habitats, different labels were used to differentiate the tumor lesion localized in the nasopharynx (A-C, label 1, green), from that invading the skull base bone and intracranial cavity (B-D, label 2, red). Segmented lesion of label 1 refers to ROI_nx; combined lesion of label 1 and 2 refers to ROI_total. For patients with synchronous nodal recurrence, additional label (A, label 3, blue) was used to delineate the lymph nodes, but it would not be used for analysis in this study. ROI, region-of-interest.

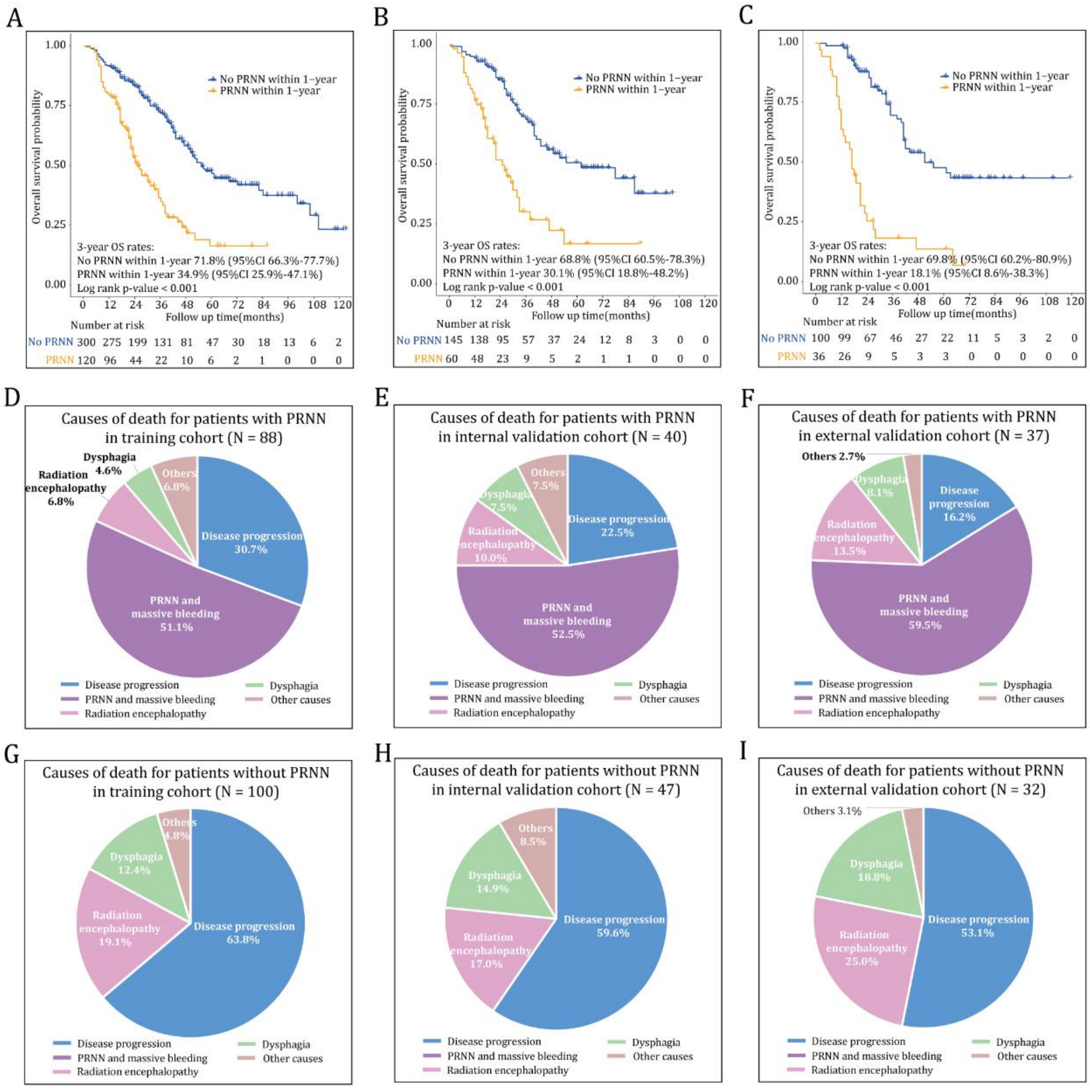

Figure S4. Overall survival of patients with and without post-radiation nasopharyngeal necrosis.

(A-C) Kaplan-Meier curves showing PRNN significantly impaired overall survival in the training (A), internal validation (B), and external validation (C) cohorts. (D-F) PRNN and massive bleeding are leading causes of death for patients with PRNN in the training (D), internal validation (E), and external validation (F) cohorts. (G-I) Disease progression is the leading cause of deaths for patients without PRNN in the training (G), internal validation (H), and external validation (I) cohorts. PRNN, post-radiation nasopharyngeal necrosis.

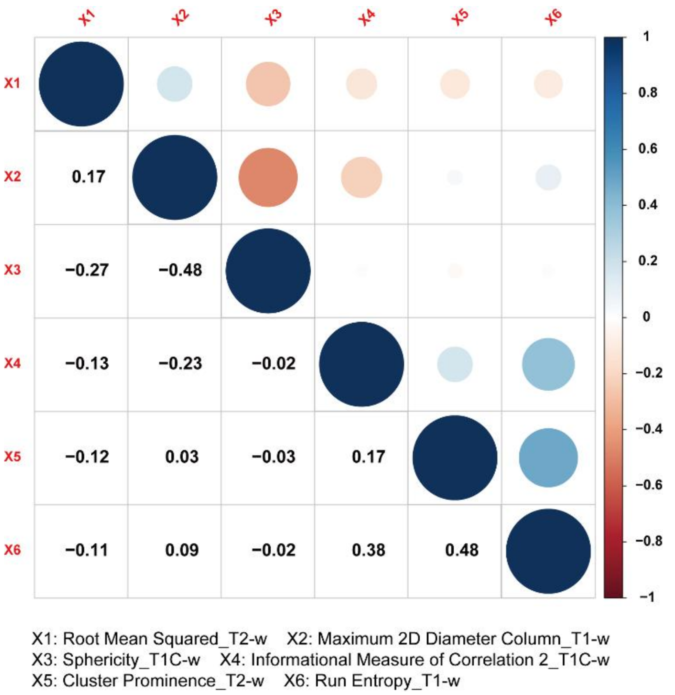

Figure S5. Pair-wise correlations among six selected radiomic features.

The correlation coefficients of Spearman test among features were all below 0.5.

Training

		Prediction		
		0	1	Sensitivity = 0.750
Reference	0	208	92	Specificity = 0.693
	1	30	90	Accuracy = 0.710

Internal validation

		Prediction		
		0	1	Sensitivity = 0.683
Reference	0	103	42	Specificity = 0.710
	1	19	41	Accuracy = 0.702

External validation

		Prediction		
		0	1	Sensitivity = 0.694
Reference	0	77	23	Specificity = 0.770
	1	11	25	Accuracy = 0.750

Figure S6. Confusion matrix for the prediction of PRNN using radiomics model.

The confusion matrix was constructed for the training, internal validation, and external validation cohorts at an optimal threshold of 0.732.

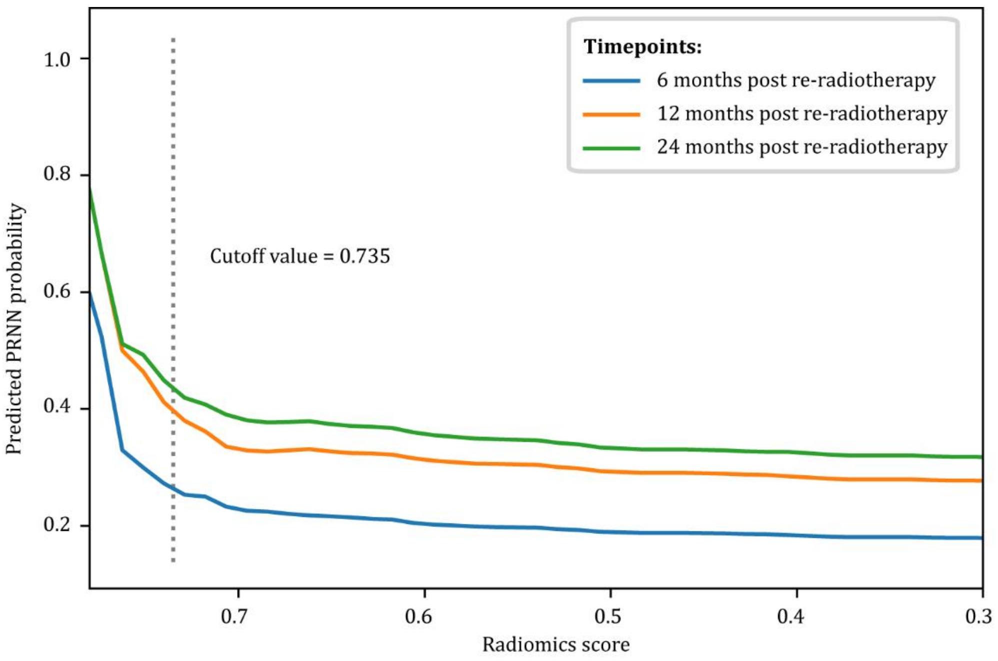

Figure S7. Nomogram predicting risk of post-radiation nasopharyngeal necrosis at different timepoints. Depending on the radiomic signature, the probability of PRNN can be indicated at a time using a nomogram. The time is defined by the color of the line (6, 12, and 24 months post re-radiotherapy). This method illustrates the value of the signature to predict PRNN in a continuous fashion as opposed to a regular Kaplan Meier that allows only discrete levels of the predictor. The PRNN probability plots were constructed with the following formula: (t)=1-e^(-H_0 (t)*e^PI), where H_0(t) is the cumulative underlying hazard function from the Cox regression and PI is the signature (predictive index). PRNN, post-radiation nasopharyngeal necrosis.

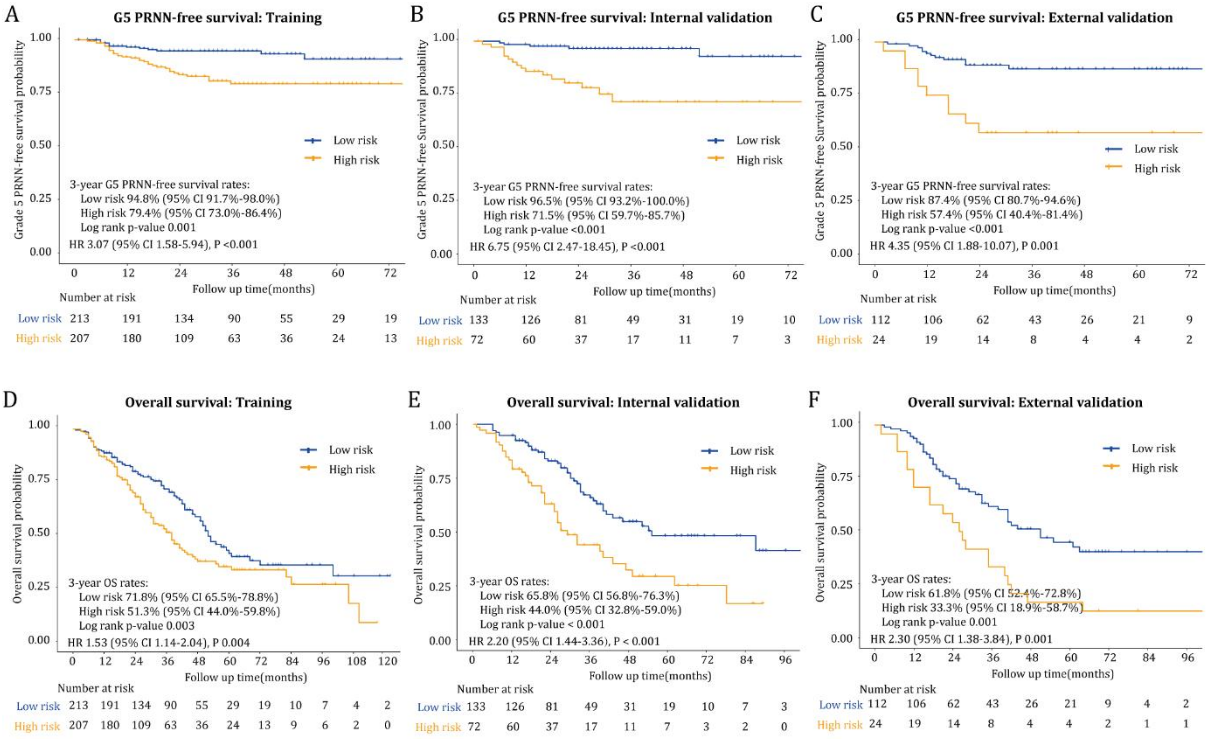

Figure S8. Prognostic significance of the radiomic signature.

(A-C) Kaplan-Meier curves of grade 5 PRNN-free survival for high risk vs low risk patients stratified by the radiomic signature in the training (A), internal validation (B) and external validation (C) cohorts. (D-F) Kaplan-Meier curves of overall survival for high risk vs low risk patients stratified by the radiomic signature in the training (D), internal validation (E) and external validation (F) cohorts. HRs were estimated using univariate Cox regression analyses. PRNN, post-radiation nasopharyngeal necrosis; OS, overall survival; HR, hazard ratio; CI, confidence interval.

Table S1. Acquisition parameters of MRI scanning.

Parameter	Sun Yat-sen University Cancer Center (SYSUCC)	Cancer Hospital Chinese Academy of Medical Sciences (CHCAMS)	Fudan University Shanghai Cancer Center (FUSCC)	Nanfang Hospital of Southern Medical University (NHSMU)
Manufacturer (mode)	GE (Discovery MR750, Discovery MR750w, Signa Excite, Signa HDx, Signa Pioneer), PHILIPS (Achieva, Panorama HFO), SIEMENS (TrioTim, Espree, Aera), and UIH (uMR 780)	GE (Optima MR360, Signa HDx)	GE (Signa HDx, Signa Voyager), and SIEMENS (Verio, Skyra)	GE (Optima MR360, Signa HDx, Signa Excite), and SIEMENS (Magnetom Vision plus, Avanto)
Magnetic Field Strength	1.5T, 3T	1.5 T	1.5T, 3T	1.5T, 3T
T1-weighted				
Repetition time	340 ms to 798 ms	308 ms to 540 ms	340 ms to 616 ms	540 ms to 896 ms
Echo time	6 ms to 15 ms	8 ms to 11 ms	7 ms to 16 ms	9 ms to 22 ms
Matrix	300*320 to 768*768	512*512	270*320 to 512*512	256*256 to 512*512
Field of view	190 mm*190 mm to 280 mm*280 mm	240 mm*240 mm to 280 mm*280 mm	210 mm*230 mm to 270 mm*270 mm	200 mm*200 mm to 250 mm*250 mm
Slice thickness	5 mm, 5.5 mm	4 mm, 5 mm	4.5 mm, 5 mm, 6 mm	5 mm, 5.5 mm, 6 mm
Slice gap	1 mm, 1.5 mm	1 mm	1 mm	1 mm, 1.2 mm, 2 mm
T1C-weighted [a]				
Repetition time	179 ms to 787 ms	280 ms to 325 ms	164 ms to 230 ms	400 ms to 600 ms
Echo time	4 ms to 17 ms	2.8 ms to 4.2 ms	1.5 ms to 2.5 ms	10 ms to 20 ms
Matrix	300*320 to 768*768	512*512	324*384 to 512*512	256*256 to 512*512
Field of view	190 mm*190 mm to 280 mm*280 mm	240 mm*240 mm to 260 mm*260 mm	220 mm*230 mm to 270 mm*270 mm	200 mm*200 mm to 250 mm*250 mm
Slice thickness	5 mm, 5.5 mm	4 mm, 5 mm	4.5 mm, 5 mm, 6 mm	5 mm, 5.5 mm, 6 mm
Slice gap	1 mm, 1.5 mm	1 mm	1 mm	1 mm, 1.2 mm, 2 mm
T2-weighted				
Repetition time	2000 ms to 9483 ms	4128 ms to 5620 ms	2500 ms to 5000 ms	2351 ms to 5000 ms
Echo time	51 ms to 125 ms	85 ms to 92 ms	65 ms to 88 ms	68 ms to 99 ms
Matrix	288*288 to 768*768	512*512	270*352 to 512*512	256*256 to 512*512
Field of view	190 mm*190 mm to 280 mm*280 mm	240 mm*240 mm to 270 mm*270 mm	210 mm*230 mm to 270 mm*280 mm	200 mm*200 mm to 250 mm*250 mm
Slice thickness	5 mm, 5.5 mm	4 mm, 5 mm	4.5 mm, 5 mm, 6 mm	5 mm, 5.5 mm, 6 mm
Slice gap	1 mm, 1.5 mm	1 mm	1 mm	1 mm, 1.2 mm, 2 mm

[a] Enhanced T1-weighted sequences were performed 40 seconds after intravenous Gd-DTPA (Magnevist; Bayer Schering Pharma AG, Germany) injection at a dose of 0.1 mmol/kg.
MRI, Magnetic Resonance Imaging.

Table S2. Summary of clinical outcomes of patients in training and validation cohorts.

Items	Training cohort	Internal validation cohort	External validation cohort
Sanple size	420	205	136
Median follow up time, months (95% CI)	44 (39-48)	39 (34-39)	48 (44-66)
Median overall survival, months (95% CI)	46 (41-51)	43 (37-78)	41 (35-61)
Overall death event (%)	188 (44.8)	87 (42.4)	69 (50.7)
PRNN event within 1-year (%)	120 (28.5)	60 (29.2)	36 (26.4)
Overall PRNN event (%)	148 (35.2)	66 (32.1)	45 (33.0)
Outcomes of patients			
Survive	232 (55.3)	118 (57.6)	67 (49.2)
Die of disease progression	89 (21.2)	37 (18.0)	23 (16.9)
Die of PRNN-related factors [a]	45 (10.7)	21 (10.2)	22 (16.2)
Die of other complications [b]	43 (6.6)	22 (10.8)	22 (16.2)
Die of unknown reasons	11 (2.6)	7 (3.4)	2 (1.5)

[a] PRNN-related factors included massive bleeding, central nervous infection, osteomyelitis, suicide and other events owing to PRNN-related complications.

[b] Other complications included dysphagia, radiation encephalopathy and massive bleeding without evidences of PRNN.

PRNN, post-radiation nasopharyngeal necrosis; CI, confidence interval.

Table S3. Choices of methods for radiomic signature development.

Classifier	Feature selection method	AUC based on ROI_nx [a]			AUC based on ROI_total [b]		
		Training cohort	Internal validation	External validation	Training cohort	Internal validation	External validation
Random Forest	Linear SVM	0.722	0.713	0.756	0.708	0.731	0.721
	Lasso regression	0.682	0.596	0.543	0.721	0.604	0.467
	Logistic regression	0.714	0.641	0.584	0.708	0.604	0.664
Linear SVM	Linear SVM	0.647	0.704	0.722	0.656	0.714	0.721
	Lasso regression	0.458	0.360	0.465	0.599	0.575	0.521
	Logistic regression	0.668	0.705	0.668	0.636	0.593	0.673
Logistic regression	Linear SVM	0.647	0.702	0.726	0.657	0.711	0.729
	Lasso regression	0.565	0.568	0.455	0.596	0.568	0.518
	Logistic regression	0.669	0.700	0.671	0.635	0.598	0.677

[a] ROI_nx refers to the segmented tumor localized in the nasopharynx.

[b] ROI_total refers to the whole tumor that includes lesions beyond the nasopharynx and invading the skull base bone and intracranial cavity.

AUC, the area under the receiver operating characteristic curve; ROI, region-of-interest; SVM, Support Vector Machines.

Table S4. Number of features remained after each step of feature selection.

Radiomic features selection step	T1-w MRI	T2-w MRI	T1C-w MRI	Overall
Before selection	702	702	702	2106
Reproducibility measurement	503	550	577	1630
SVM for feature importance	100	100	100	300
RFE for feature selection and uncorrelation	2	2	2	6

MRI, Magnetic Resonance Imaging; SVM, Support Vector Machines; RFE, recursive feature elimination.

Table S5. Details of the six radiomic features included in the random forest model.

ID	Name	Type	Image	MRI sequence	Annotation
feature 3	Root Mean Squared	first order	LoG filter, sigma 3mm	T2-w	Intensity
feature 1	Sphericity	shape	original	T1C-w	Sphericity
feature 2	Maximum 2D Diameter Column	shape	original	T1-w	Size
feature 4	Run Entropy of GLRLM	texture	wavelet, HL	T1-w	Heterogeneity
feature 5	Informational Measure of Correlation 2 of GLCM	texture	wavelet, HL	T1C-w	Complexity
feature 6	Cluster Prominence of GLCM	texture	LoG filter, sigma 1mm	T2-w	Skewness and asymmetry

MRI, Magnetic Resonance Imaging; GLRLM, Gray Level Run-Length Matrix; GLCM, Gray Level Co-occurrence Matrix; H: high wavelet filter; L: low wavelet filter.

Table S6. Candidate clinical and radiomics variables for prediction of PRNN in training cohort.

Variable	AUC for 1-year PRNN (95% CI)	Cutoff value determination	HR for PRNN (95% CI)	P value
PRNN radiomic signature (> 0.735 vs ≤ 0.735)	0.722 (0.676-0.765)	Best log-rank	2.53 (1.80-3.56)	<0.001
Age (> 45 years vs ≤ 45 years)	0.565 (0.507-0.623)	Youden index	1.43 (1.03-2.00)	0.034
Sex (female vs male)	0.519 (0.472-0.566)	Clinical	1.26 (0.88-1.80)	0.217
BMI (> 21.3 kg/m2 vs ≤ 21.3 kg/m2)	0.492 (0.432-0.552)	Youden index	1.13 (0.80-1.58)	0.490
DFI (> 37 months vs ≤ 37 months)	0.552 (0.492-0.613)	Youden index	1.28 (0.93-1.77)	0.136
Recurrent T category (rT3-4 vs rT1-2)	0.498 (0.438-0.558)	Clinical	0.71 (0.48-1.05)	0.085
$GTV_{recurrence}$ (> 37.3 cm3 vs ≤ 37.3 cm3)	0.588 (0.530-0.646)	Youden index	1.64 (1.17-2.29)	0.004
Re-irradiation dose (> 61.1 Gy vs ≤ 61.1 Gy)	0.496 (0.438-0.554)	Median	1.14 (0.81-1.59)	0.459
Accumulated GTV dose (> 131.1 Gy vs ≤ 131.1 Gy)	0.494 (0.437-0.551)	Median	1.14 (0.82-1.59)	0.439
Combination with chemotherapy (yes vs no)	0.458 (0.408-0.509)	Clinical	0.81 (0.57-1.14)	0.220
Hemoglobin level (> 137 g/L vs ≤ 137 g/L)	0.440 (0.378-0.502)	Median	0.81 (0.58-1.12)	0.194

PRNN, post-radiation nasopharyngeal necrosis; BMI, Body Mass Index; GTV, gross tumor volume; DFI, disease-free interval; AUC, area under the receiver operating characteristic curve; HR, hazard ratio; CI, confidence interval.

Table S7. Performance of the radiomics and clinical models in training and validation cohorts.

Model	Training cohort		Internal validation cohort		External validation cohort	
	AUC (95% CI)	P value	AUC (95% CI)	P value	AUC (95% CI)	P value
Radiomic score	0.722 (0.676-0.765)	referent	0.713 (0.653-0.772)	referent	0.756 (0.673-0.838)	referent
Clinical score	0.600 (0.543-0.658)	0.001	0.604 (0.519-0.689)	0.031	0.571 (0.449-0.692)	0.005
Radiomic plus re-irradiation dose	0.741 (0.697-0.783)	0.096	0.717 (0.657-0.780)	0.760	0.762 (0.676-0.844)	0.550
Radiomic plus clinical	0.720 (0.675-0.760)	0.878	0.733 (0.670-0.792)	0.265	0.720 (0.627-0.803)	0.083

[a] Clinical score was developed based on five clinical factors including sex, age, disease-free interval, $GTV_{recurrence}$ and re-irradiation dose.

[b] Performance of the models were compared with DeLong test.

PRNN, post-radiation nasopharyngeal necrosis; AUC, area under the receiver operating characteristic curve; CI, confidence interval.

Table S8. Generalizability of the radiomic signature across medical centers and MRI parameters.

MRI parameters	Sample size	AUC (95%CI)
Medical center		
SYSUCC	625 (82.1)	0.716 (0.673-0.759)
CHCAMS	37 (4.9)	0.888 (0.785-0.991)
FUSCC	77 (10.1)	0.671 (0.521-0.820)
NHSMU	22 (2.9)	0.865 (0.669-1.000)
Manufacturer		
GE Medical Systems	375 (49.3)	0.718 (0.661-0.774)
Philips Medical Systems	192 (25.2)	0.713 (0.632-0.794)
SIEMENS	163 (21.4)	0.728 (0.646-0.809)
UIH	31 (4.1)	0.803 (0.636-0.970)
Magnetic Field Strength		
1.5 T	281 (36.9)	0.728 (0.661-0.794)
3.0 T	480 (63.1)	0.715 (0.665-0.764)
Slice thickness		
4.0-5.0 mm	666 (87.5)	0.715 (0.673-0.757)
5.5-6.0 mm	95 (12.5)	0.707 (0.560-0.853)
Slice gap		
1.0 mm	713 (93.7)	0.718 (0.677-0.759)
1.2-2.0 mm	48 (6.3)	0.813 (0.690-0.936)

MRI, Magnetic Resonance Imaging; AUC, area under the receiver operating characteristic curve; CI, confidence interval; SYSUCC, Sun Yat-sen University Cancer Center; CHCAMS, Cancer Hospital Chinese Academy of Medical Sciences; FUSCC, Fudan University Shanghai Cancer Center; NHSMU, Nanfang Hospital of Southern Medical University.

Table S9. Generalizability of the radiomic signature across patient subgroups.

Clinical variables	Sample size (%)	PRNN within 1-year (%)	AUC (95% CI)	1-year PRNN rate in low-risk group	1-year PRNN rate in high-risk group	HR (95% CI)	P value
Age							
≤ 45 years	308 (40.5)	74 (24.0)	0.695 (0.627-0.763)	16.2%	36.9%	2.05 (1.36-3.10)	0.001
> 45 years	453 (59.5)	142 (31.3)	0.738 (0.690-0.786)	18.3%	51.8%	3.09 (2.26-4.23)	< 0.001
Sex							
Male	566 (74.4)	154 (27.2)	0.709 (0.663-0.755)	16.7%	44.5%	2.60 (1.94-3.48)	< 0.001
Female	195 (25.6)	62 (31.8)	0.752 (0.678-0.826)	19.7%	49.5%	2.81 (1.76-4.49)	< 0.001
BMI							
≤ 21.3 kg/m2	293 (38.5)	80 (27.3)	0.680 (0.613-0.746)	18.7%	40.4%	2.36 (1.59-3.51)	< 0.001
> 21.3 kg/m2	468 (61.5)	136 (29.1)	0.748 (0.700-0.796)	16.7%	49.5%	2.89 (2.10-3.97)	< 0.001
Recurrent T category							
rT1-2	148 (19.4)	39 (26.4)	0.809 (0.732-0.886)	18.0%	65.4%	4.03 (2.26-7.20)	< 0.001
rT3-4	613 (80.6)	177 (28.9)	0.705 (0.660-0.750)	17.2%	43.9%	2.51 (1.90-3.33)	< 0.001
Synchronous nodal recurrence							
No	526 (69.1)	150 (28.5)	0.713 (0.665-0.761)	17.8%	46.0%	2.72 (2.02-3.66)	< 0.001
Yes	235 (30.9)	66 (28.1)	0.743 (0.675-0.811)	16.6%	45.4%	2.59 (1.64-4.07)	< 0.001
$GTV_{recurrence}$							
≤ 37.3 cm3	376 (49.4)	85 (22.6)	0.701 (0.638-0.763)	17.0%	40.4%	2.30 (1.56-3.40)	< 0.001
> 37.3 cm3	385 (50.6)	131 (34.0)	0.712 (0.660-0.765)	18.2%	48.3%	2.72 (1.91-3.88)	< 0.001
Disease-free interval							
≤ 37 months	409 (53.7)	99 (24.2)	0.699 (0.641-0.758)	16.5%	38.5%	2.47 (1.73-3.52)	< 0.001
> 37 months	352 (46.3)	117 (33.2)	0.739 (0.685-0.792)	18.8%	53.1%	2.85 (2.00-4.05)	< 0.001
Re-irradiation dose							
≤ 61.11 Gy	280 (36.8)	77 (27.5)	0.765 (0.704-0.826)	13.3%	44.7%	3.52 (2.26-5.49)	< 0.001
> 61.11 Gy	481 (63.2)	139 (28.9)	0.703 (0.653-0.753)	19.5%	46.7%	2.34 (1.72-3.18)	< 0.001
Accumulated GTV dose							
≤ 131.11 Gy	306 (40.2)	79 (25.8)	0.794 (0.738-0.849)	11.6%	44.9%	4.36 (2.77-6.86)	< 0.001
> 131.11 Gy	455 (59.8)	137 (30.1)	0.684 (0.633-0.736)	21.0%	46.6%	2.09 (1.54-2.83)	< 0.001
Combination with chemotherapy							
No	242 (31.8)	71 (29.3)	0.735 (0.669-0.802)	19.5%	46.9%	2.63 (1.70-4.06)	< 0.001
Yes	519 (68.2)	145 (27.9)	0.718 (0.670-0.766)	16.4%	45.4%	2.68 (1.99-3.63)	< 0.001
Hemoglobin level							
≤ 137 g/L	398 (52.3)	123 (30.9)	0.723 (0.670-0.776)	18.8%	47.6%	2.35 (1.70-3.24)	< 0.001
> 137 g/L	363 (47.7)	93 (25.6)	0.717 (0.659-0.776)	16.1%	43.7%	3.05 (2.07-4.48)	< 0.001

PRNN, post-radiation nasopharyngeal necrosis; BMI, Body Mass Index; GTV, gross tumor volume; AUC, area under the receiver operating characteristic curve; HR, hazard ratio; CI, confidence interval.

Table S10. Prognostic significance of the radiomic signature in multivariate Cox analyses.

Variables	Training		Internal validation		External validation	
	HR (95% CI)	P value	HR (95% CI)	P value	HR (95% CI)	P value
PRNN						
PRNN radiomic signature (high-risk vs low-risk)	2.41 (1.68-3.44)	< 0.001	2.77 (1.66-4.62)	< 0.001	3.39 (1.65-6.97)	< 0.001
Age (> 45 years vs ≤ 45 years)	1.39 (0.99-1.94)	0.057	1.33 (0.76-2.32)	0.318	1.02 (0.54-1.91)	0.960
Sex (female vs male)	1.33 (0.93-1.92)	0.121	1.48 (0.86-2.53)	0.155	0.80 (0.40-1.59)	0.522
$GTV_{recurrence}$ (> 37.3 cm3 vs ≤ 37.3 cm3)	1.25 (0.88-1.78)	0.208	1.16 (0.68-1.97)	0.588	1.10 (0.55-2.20)	0.797
DFI (> 37 months vs ≤ 37 months)	1.17 (0.84-1.63)	0.349	1.30 (0.77-2.20)	0.317	1.21 (0.65-2.24)	0.553
Re-irradiation dose (> 61.1 Gy vs ≤ 61.1 Gy)	1.26 (0.90-1.77)	0.183	0.68 (0.41-1.11)	0.125	0.95 (0.44-2.02)	0.889
G5 PRNN-free survival						
PRNN radiomic signature (high-risk vs low-risk)	2.61 (1.31-5.20)	0.006	5.24 (1.85-14.88)	0.002	4.45 (1.66-11.89)	0.003
Age (> 45 years vs ≤ 45 years)	1.29 (0.70-2.38)	0.407	1.25 (0.47-3.29)	0.656	0.83 (0.35-1.96)	0.667
Sex (female vs male)	1.00 (0.49-2.02)	0.993	2.35 (0.89-6.26)	0.086	0.81 (0.30-2.19)	0.675
$GTV_{recurrence}$ (> 37.3 cm3 vs ≤ 37.3 cm3)	2.89 (1.36-6.17)	0.006	3.77 (1.26-11.3)	0.018	1.19 (0.44-3.18)	0.731
DFI (> 37 months vs ≤ 37 months)	0.78 (0.43-1.42)	0.413	0.69 (0.27-1.75)	0.435	0.87 (0.36-2.10)	0.749
Re-irradiation dose (> 61.1 Gy vs ≤ 61.1 Gy)	1.95 (1.00-3.81)	0.050	0.40 (0.16-0.97)	0.043	0.63 (0.24-1.68)	0.356
Overall survival						
PRNN radiomic signature (high-risk vs low-risk)	1.37 (1.00-1.86)	0.049	1.94 (1.25-3.02)	0.003	1.88 (1.02-3.45)	0.043
Age (> 45 years vs ≤ 45 years)	1.13 (0.85-1.52)	0.398	1.39 (0.86-2.25)	0.176	1.44 (0.86-2.41)	0.164
Sex (female vs male)	1.07 (0.77-1.50)	0.679	0.99 (0.59-1.68)	0.981	0.73 (0.42-1.27)	0.262
$GTV_{recurrence}$ (> 37.3 cm3 vs ≤ 37.3 cm3)	1.67 (1.22-2.28)	0.001	1.83 (1.14-2.92)	0.012	1.58 (0.92-2.73)	0.097
DFI (> 37 months vs ≤ 37 months)	0.81 (0.60-1.09)	0.162	0.69 (0.44-1.09)	0.115	0.99 (0.59-1.66)	0.974
Re-irradiation dose (> 61.1 Gy vs ≤ 61.1 Gy)	1.04 (0.77-1.41)	0.804	0.73 (0.47-1.12)	0.151	0.98 (0.53-1.79)	0.940

PRNN, post-radiation nasopharyngeal necrosis; BMI, Body Mass Index; GTV, gross tumor volume; DFI, disease-free interval; HR, hazard ratio; CI, confidence interval.

Table S11. Core enrichment genes of the biological processes associated with the radiomic signature.

Gene set	Normalized enrichment score	Nominal P value	Core enrichment genes
GOBP: REGULATION OF FIBROBLAST PROLIFERATION	-1.58	0.030	SKI, MED25, PAWR, CD300A, TRIM32, DAB2IP, CDC73, TP53, PARP10, MORC3
GOBP: FIBROBLAST MIGRATION	-1.50	0.049	FGF2, PDLIM1, RCC2, CORO1C, APPL2, TMEM201, TGFB1, PAK3, ZFAND5, ADIPOR2, APPL1, SLC8A1, ARID5B, PRKCE, FER, MTA2, RFFL, SGPL1, PAK1, PTK2 PML, BAG4
GOBP: FIBROBLAST APOPTOTIC PROCESS	-1.71	0.004	API5, PIK3CA, STK17B, CHD8, STK17A, CUL3, PRDM11, TP63, BTG1, TP53, BCL2L11
GOBP: POSITIVE REGULATION OF LEUKOCYTE ADHESION TO VASCULAR ENDOTHELIAL CELL	-1.78	<0.001	ETS1, ELANE, RHOA, NFAT5, FUT7, ALOX5, PTAFR, ITGB2, ICAM1, TRAF6, RELA
GOBP: REGULATION OF ENDOTHELIAL CELL APOPTOTIC PROCESS	-1.50	0.021	GATA3, PAK4, GATA2, PDPK1, ANGPT1, MIR30E, ABL1, NFE2L2, SEMA5A, IL13, ICAM1, TNFAIP3
GOBP: VASCULAR ENDOTHELIAL GROWTH FACTOR RECEPTOR SIGNALING PATHWAY	-1.46	0.019	NCF2, CRK, NIBAN2, FGF18, MAPKAPK2, MMRN2, ITGB3, IL1B, PRKD2, MAPK14, ELMO2, CNMD, PTK2B, PRKCB, SRC, HIF1A, PAK2, NCF4, FYN, CYFIP2, VEGFA, PIK3R1, EPN2, ROCK1, PIK3CA, RHOA, CDC42, FOXC2, FOXC1, SHB, NCKAP1L, NEDD4, ARNT, NCK1, DOCK1, PTPN1, HGS, CYBB, DAB2IP, VAV2, ITGAV, WASF2, AXL, BAIAP2, PIK3R2, NCF1, PTK2, PIK3CB, CYFIP1, ABI1, MAPKAPK3, SH2D2A
